# Exploring the Potential and Evaluating Hydrocarbon Degradation by Novel Antarctic *Dietzia* and *Pusillimonas* Isolates From a Pristine Environment

**DOI:** 10.1111/1758-2229.70248

**Published:** 2026-01-08

**Authors:** Tomasz Krucoń, Julia Karbowska, Wiktoria Pietrowicz, Robert Stasiuk, Łukasz Drewniak

**Affiliations:** ^1^ Department of Environmental Microbiology and Biotechnology, Faculty of Biology Institute of Microbiology, University of Warsaw Warsaw Poland; ^2^ Department of Geomicrobiology, Faculty of Biology Institute of Microbiology, University of Warsaw Warsaw Poland; ^3^ Department of Microbiology, Faculty of Natural Sciences Institute of Biology, Jan Kochanowski University Kielce Poland

**Keywords:** bioaugmentation, bioremediation, *Dietzia*, phylogenomics, *Pusillimonas*, resistance

## Abstract

This study evaluated the bioremediation potential of two psychrotolerant strains, *Pusillimonas* sp. ANT_WB101 and *Dietzia* sp. ANT_WB102, isolated from an uncontaminated water pond. Comparative analysis indicated affinities with *P. gingsengisoli/soli* and *D. kunjamensis/maris* . Both strains demonstrated substantial crude oil degradation efficiency, achieving ≥ 79% under aerobic conditions and ≥ 34% under anaerobic conditions. Genomic analysis identified crucial genes involved in crude oil degradation, including alkane monooxygenase, cytochrome P450, and polyphenol monooxygenase. These strains displayed adaptability to a wide range of environmental factors, such as pH (4–11), salinity (up to 6%–9%), temperature (4°C–37°C), resistance to freeze–thaw cycles, and tolerance to high crude oil concentration. Biosafety evaluations indicated the sensitivity of the strains to various antibiotics, ensuring their suitability and safety for environmental applications. At low temperature, these strains increased microbial activity in environmental samples (1.34 times in biological oxygen demand compared to the control) and showed effective biodegradation (~39%). In conclusion, the study highlights the potential of ANT_WB101 and ANT_WB102 for treating contaminated sites and indicates the possible challenges of using microorganisms not initially adapted to site‐specific conditions in bioremediation efforts.

## Graphical abstract

The Antarctic *Dietzia* and *Pusillimonas* isolates were tested for bioremediation applications. Genomic analyses revealed the potential to degrade complex organic compounds, potential virulence factors and resistance genes. Physiological assays showed a wide range of adaptation to abiotic factors and sensitivity to different groups of antibiotics. Functional tests confirmed the ability to degrade hydrocarbons, and further tests in microcosms indicated the influence of the analysed bacteria on clean‐up processes.

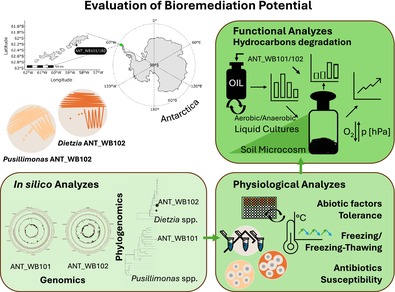

## 1 Introduction

In natural environment, certain microorganisms possess the ability to transform or mineralise contaminants, generally resulting in the formation of less harmful substances that may be incorporated into natural biogeochemical cycles (Doukani et al. 2022). Petroleum hydrocarbons are among these contaminants and represent one of the most significant sources of soil pollution (Panagos et al. 2013). Hydrocarbon contaminations, caused by industrial activities and anthropogenic sources, remains a pressing global issue due to its harmful effects on ecosystems and human health (Mallah et al. 2022; Davidson et al. 2021; McKee et al. 2018). In this context, bioremediation, utilising the metabolic capabilities of microorganisms to degrade hydrocarbons, has emerged as a sustainable approach for mitigating these pollutants and restoring the environment (Singh et al. 2020).

Although consortia of microorganisms isolated from polluted environments showed promising results in bioremediation (Bai et al. 2017; Patowary et al. 2016; Janbandhu and Fulekar 2011; Vasudevan and Rajaram 2001), exploring novel bacterial strains from extreme, uncontaminated ecosystems provides an intriguing opportunity. The isolation and characterisation of these strains provide fresh insights into the natural abilities of microorganisms in contaminants degradation. In a previous study (Krucon et al. 2021), we examined the metabolic capabilities of bacterial communities from a periodic water pond on King George Island, including the degradation of acyclic hydrocarbons. Of the identified strains *Dietzia* and *Pusillimonas* showed the most promise. These bacterial genera, representing Actinomycetota and Pseudomonadota, respectively, have not been extensively studied compared to more well‐known strains. However, there has been an increasing interest in these microorganisms in recent years due to their versatile metabolism and unique genomic traits that contribute to their survivability in various environments, from cold environments (Ausuri et al. 2021; Papale et al. 2022; Rogala et al. 2020; Mayilraj et al. 2006) to hydrothermal vents (Wang et al. 2020), and from the rhizosphere (Bharti et al. 2016) to activated sludge (Song et al. 2022; Yao et al. 2022; Koh et al. 2019), as well as in human clinical specimens (Brown et al. 2022; Kämpfer et al. 2012). Their ability to degrade petroleum derivatives is of particular interest (Li et al. 2013; Wang et al. 2011; Hilyard et al. 2008; von der Weid et al. 2007). Further research on novel isolates, *Dietzia* and *Pusillimonas*, from harsh environments could provide more significant insight into their potential contributions to hydrocarbon degradation in challenging environments, particularly in cold temperatures.

In this study we conducted a comprehensive analysis of two Antarctic strains, *Dietzia* sp. ANT_WB102 and *Pusillimonas* sp. ANT_WB101 with an emphasis on their application in hydrocarbon degradation. Our examination of the physiological and genomic traits of these bacteria showed remarkable adaptation, enabling them to thrive in a broad range of abiotic factors. Exploration of their applicability in environmental treatment demonstrated a high efficiency in degrading crude oil, while bioaugmentation of non‐sterile environmental samples revealed important aspects to consider in bioremediation. Considering the distinctive nature of their origins, comprehensive phylogenomic evaluations established their taxonomic position and similarity to other bacteria within the respective genera. The potential utility of these isolates required exploring the virulence factors and antibiotic resistance as well, indicating their safety in environmental applications. The outcomes of these studies highlight the promising potential of Antarctic isolates for environmental treatment and offer a significant contribution to the ongoing efforts to restore environments impacted by hydrocarbon pollutants.

## 2 Materials and Methods

### 2.1 Strains and Culture Condition

The studied strains were previously isolated from a freshwater pond located on King George Island (62°14′4.76″ S, 58°28′22.29″ W), part of the South Shetland Islands, near the Antarctic Peninsula and approximately 8.3 km from the Polish Arctowski Station (Krucon et al. 2021). The average air temperature at the station during the austral summer was approximately 2°C–2.4°C, whereas the mean annual air temperature on the island was around −1.2°C. The mean ground temperature at a depth of 5–70 cm remains close to 0.4°C throughout the year (Araźny et al. 2013). Liquid bacteria cultures were prepared by inoculating a loop of cells from a plate in 30 mL of Luria Broth medium (LB Broth, BioMaxima) within 100 mL Erlenmeyer flasks and incubated at room temperature (22°C) under 180 rpm agitation for 72 h. Subsequently, the bacterial culture was directly inoculated into a medium. Unless otherwise specified, the reagents were sourced from Sigma–Aldrich or Merck.

### 2.2 Resistance to Environmental Factors

Bacterial tolerance to temperature (4°C–37°C), pH (2–12) and salinity (0–100 g L^−1^) was tested by measuring the culture's optical density at 600 nm in 96‐well plates in LB. pH adjustments were made with NaOH (Chempur) and HCl, while salinity was established using NaCl. Cultures were agitated for 5 days at room temperature at 180 rpm, and photometric measurements at 600 nm wavelength were performed using the Sunrise TM. Heavy metals and petroleum tolerance, as well as resistance to freezing and freeze–thaw cycles were evaluated based on the methodology described in Krucoń et al. (2023). Briefly, metal concentrations in LB medium ranged from 0.01 to 500 mM, and survivability was measured using 96‐well plates at 600 nm. For petroleum survivability, bacteria were quantified after incubation in soil samples with 1000–20,000 mg kg^−1^ of dry weight (DW). Freeze–thaw resistance was determined by counting bacterial colonies after thawing frozen cultures at 4°C (previously stored at −20°C). All experiments were performed in triplicate with three technical replicates for 96‐well plate tests.

### 2.3 Nitrate Respiration Assay

Nitrate respiration was tested using a medium composed of 0.61 g L^−1^ K_2_KPO_4_, 0.2 g L^−1^ KH_2_PO_4_, 0.1 g L^−1^ CaCl_2_, 0.5 g L^−1^ MgSO_4_ × 7H_2_O (VWR Chemicals), 1.5 g L^−1^ (NH_4_)_2_SO_4_, 3.0 g L^−1^ yeast extract (BLT), 100 mg L^−1^ KNO_3_, 10 g L^−1^ glycerine (VWR Chemicals) (modified, according to Stolp and Gadkari 1981). Cultures were grown in 80 mL of the medium in 100 mL sealed bottles and flushed with a CO_2_:N_2_ mixture. The cultures were then agitated at 180 rpm at 22°C for 48 h. The nitrate and nitrite content were monitored using the Nanocolor Nitrate 250, Nitrate 50 and Nitrite 4 kit.

### 2.4 Biosurfactant Production

The ability to produce biosurfactants from a low‐cost organic source was determined using an oil‐spreading assay (Morikawa et al. 2000). Bacterial cultures were cultivated in a Bushnell‐Haas medium (Bushnell and Haas 1941) with glycerol (1% w v^−1^) or molasses (1% w v^−1^) and vegetable (canola) oil. The surfactants' presence was monitored daily for 7 days by examining the appearance of oil‐free zones when the cultures were applied to a Petri dish filled with water and diesel oil.

### 2.5 Antibiotic Resistance Assessment

The ANT_WB101 susceptibility to antibiotics such as amoxicillin‐clavulanic acid, ampicillin, aztreonam, cefepime, cefuroxime, ciprofloxacin, colistin, fosfomycin, gentamicin, imipenem, meropenem, neomycin, piperacillin/tazobactam, trimethoprim, and trimethoprim/sulfamethoxazole was evaluated using the disk‐diffusion method. Meanwhile, the ANT_WB102 antibiotic resistance against ampicillin, cefotaxime, ceftriaxone, chloramphenicol, clindamycin, erythromycin, gentamicin, linezolid, streptomycin, teicoplanin, tetracycline, tigecycline, trimethoprim, and vancomycin was assessed. The evaluation was carried out on both LB and tryptic soy agar (TSA) growth media. Incubation occurred at temperatures of 22°C and 30°C for 48 h. The results were obtained after the 30°C incubation on TSA medium, as this condition demonstrated better bacterial growth. The tests were performed in duplicate.

### 2.6 Determination of Degradation Rates in Batch and Microcosms Experiments

The ability to degrade petroleum hydrocarbons was assessed in modified BH (5 g L^−1^ NH_4_NO_3_) with 1% (w v^−1^) crude oil and a trace element solution (Tuovinen et al. 1980). Experiments were conducted under aerobic and anaerobic conditions. The cultures were prepared in 30 mL volumes within 100 mL Erlenmeyer flasks, or 80 mL of medium was used in 100 mL sealed bottles and flushed with a CO_2_:N_2_ mixture, respectively. Inoculum was prepared from three‐day‐old cultures, centrifuged at 6000 rpm, and washed with a saline solution (three times). The cultures were incubated for 2 weeks at 180 rpm at 22°C. At the end of the experiment, the residual crude oil was determined by weight measurements. The control variant without bacteria was treated in the same way as the experimental groups.

Bacteria activity and their impact on the degradation rate of petroleum compounds were tested using the OxiTop system. OxiTop measures the pressure changes in closed vessels, indicating oxygen consumption through aerobic metabolism. Biostimulated and bioaugmented soil with the specified strain was used as the test sample, while biostimulated soil was used as the control. In 2.5 L vessels, 200 g of non‐sterile environmental and industrial soil contaminated with hydrocarbons were placed and bacteria suspended in BH, or sterile BH were applied to obtain a soil moisture of 20%. Bacterial counts in the inoculum were adjusted to 10^6^ CFU g^−1^ DW after soil inoculation. After 28 days of incubation at 4°C or 22°C, measurements of pressure changes were calculated according to Vähäoja et al. (2005). The experiments were conducted in duplicate.

### 2.7 Extraction and Analysis of Organic Compounds

Crude oil content in liquid cultures was determined after centrifuging the cultures in 50 mL Corning tubes at 11,000 rpm. Then, organic compounds extraction from the supernatant was carried out using a 1:1 supernatant‐to‐solvent ratio in a separatory funnel with a mixture of chloroform and ethyl acetate (3:1). After the biomass removal, residual crude oil from the glass vessels and tubes was washed and extracted three times. The resulting extract was dried with anhydrous Na_2_SO_4_, followed by solvent evaporation using a rotary evaporator, and the remaining crude oil fraction was quantified by weight. Organic compounds content including metabolites within the soil was determined through GC–MS. Samples (3–5 g each) were dried with anhydrous Na_2_SO_4_ and extracted using 150 mL of chloroform in a Soxhlet apparatus. The excess solvent was evaporated using an evaporator and the extraction solution was stored at 4°C until further analysis. For organic compounds separation, an Agilent 7890A Series Gas Chromatograph interfaced with an Agilent 5973c Network Mass Selective Detector and an Agilent 7683 Series Injector (Agilent Technologies, USA) was used, as described in Krucoń et al. (2023). The chromatograms were subsequently analysed and compared using MZmine 3 (Schmid et al. 2023) (mass detection: mass detector—centroid, ADAP Chromatogram Builder: m/z tolerance [scan‐to‐scan 0.75 m/z], ADAP resolver: S/N estimator—Wavelet coeff. SN, spectral deconvolution: hierarchical clustering, alignment: ADAP aligner, gap filling: peak finder). The resulting data, in the form of aligned and grouped peaks, were cross‐referenced with ChemStation analysis results and annotated accordingly. Biodegradation efficiency (%) was calculated according to the modified formula presented in the (Dussán and Numpaque 2012): Biodegradation efficiency = 100 − (*A*_*s*_ × 100/*A*_*ac*_), where *A*_*s*_ is the total peak area in each test sample and *A*_*ac*_ is the total peak area in the corresponding abiotic control. Total peak areas were normalised by considering the extract weight and the weight and moisture content of the soil samples.

### 2.8 Bioinformatics Analysis

Bacterial genes assignment to the appropriate ortholog groups and COGs was performed using OrthoFinder (v.2.5.5) (Emms and Kelly 2019) and the eggNOG tool (Cantalapiedra et al. 2021), respectively. *Dietzia* and *Pusillimonas* genomes available at NCBI (date of access: 24.04.2023) were chosen for comparative analysis. The common genes of the compared strains, obtained by an all‐to‐all comparison performed using blastn software (Camacho et al. 2009) and clustering based on the Markov Cluster Algorithm (Enright et al. 2002), were selected for phylogenomic analysis. The phylogenomic trees were constructed using the MEGA11 software (Tamura et al. 2021) with the Maximum Likelihood method and General Time Reversible model. All positions containing gaps and missing data were excluded. Genomic analysis in terms of the presence of genes related to the biochemical potential of bioremediation was performed using BlastKOALA (Kanehisa et al. 2016), RAST (Aziz et al. 2008) and PROKKA (Seemann 2014). Virulence determinants, antibiotic resistance, and prophages were retrieved using the Virulence Factor Database (VFDB) (Chen et al. 2005), the Resistance Gene Identifier (RGI) (Alcock et al. 2022), and Phigaro (v.2.3.0) (Starikova et al. 2020). Statistical analysis was performed using the R package, analysis of variance (ANOVA), and Tukey's post hoc tests (*α* = 0.05). Principal component analysis was performed using the phyloseq package (v.1.36.0) (McMurdie and Holmes 2013), and correlation analysis was carried out using microViz (v.0.10.5) (Barnett et al. 2021). Classification of organic compounds was performed using the ClassyFire (Djoumbou Feunang et al. 2016) tool available at http://classyfire.wishartlab.com/.

## 3 Results and Discussion

### 3.1 Genomic Features, Comparative and Phylo‐Genomics of the Strains

Genome draft analysis of *Pusillimonas* sp. ANT_WB101 and *Dietzia* sp. ANT_WB102 showed that they consist of 4.9 Mbp and 3.3 Mbp of chromosomal DNA, respectively (Table S1). Prophage regions were identified in both genomes (two in ANT_WB101 and one in ANT_WB102) containing only auxiliary genes involved in the metabolism and virulence of phages. The most similar 16S rDNA sequences belong to *P*. sp. 2083 isolated from a freshwater microbial mat (Jasnorzewski Gardens, King George Island) and *D. kunjamensis* 313 from a water evaporation tank (Belarus) (Table S2).

**FIGURE 1:**
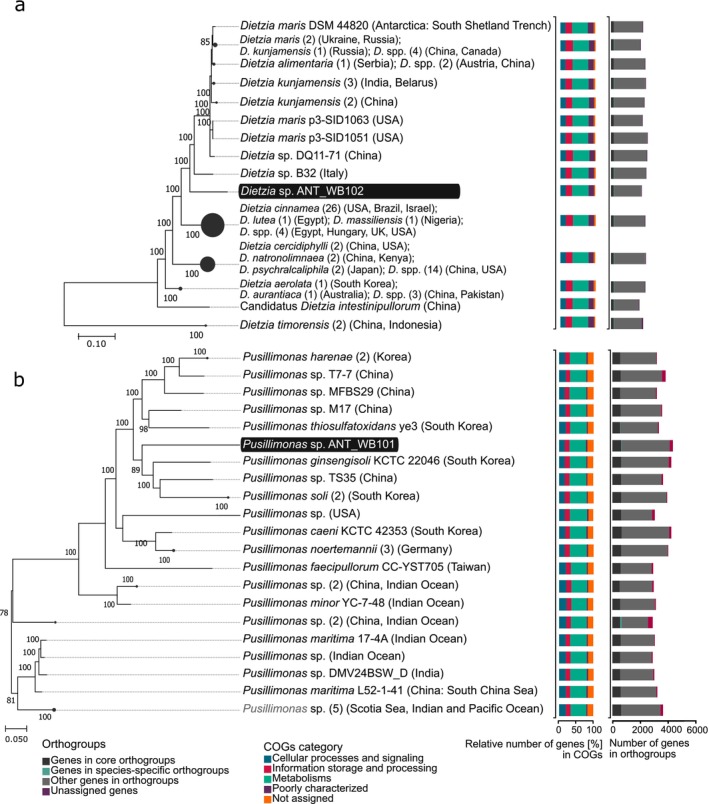
Phylogenomics trees of *Dietzia* (a) and *Pusillimonas* (b) strains, with a comparison of COGs and orthogroups. Evolutionary histories were inferred by using the Maximum Likelihood method and General Time Reversible model. A detailed description is included in the Supporting Information.

The comparative analysis with other genomes from the same genera (Figure S1), based on the nucleotide sequences of conserved genes, showed that ANT_WB101 clusters with bacteria belonging to *Pusillimonas soli* and *P. ginsengisoli* , whereas ANT_WB102 represents the outgroup of *Dietzia maris* and *D. kunjamensis* (Figure 1). In the ANT_WB101 genome, 94.8% of genes were assigned to orthogroups (96.65% in other examined *Pusillimonas* spp.), of which 0.9% were genes in species‐specific orthogroups (0.46% in others) and 17.7% of genes in core orthogroups (17.6% in others). In the case of the ANT_WB102 strain, 98.6% of genes were assigned to orthogroups (99.09% in other *Dietzia* spp.), 0.1% to species‐specific orthogroups (0.07% in others) and 8.36% to core orthogroups (7.98% in other strains).

Functional classification of COGs indicated the following categories: (1) cellular processes and signalling, (2) information storage and processing and (3) metabolism. In the case of ANT_WB101, 696 proteins encoded by genes (PEGs) (629 ± 74 in other *P*. spp.) were assigned, respectively; 556 (446 ± 74) and 2034 (1563 ± 244). In the case of ANT_WB102 443 PEGs (484 ± 49 in other *D*. spp.) were assigned to the first group, 562 (630 ± 58) to the second group and 1398 (1439 ± 129) to the last category. In the case of ANT_WB101, significantly different results compared to the other strains were obtained for the second (COGs: A and L) and third categories (E and H) and in the case of ANT_WB102 for the first category (COGs: D, U, Z), indicating different adaptive strategies.

The considerably higher number of unique genes observed in analysed isolates may be a result of the harsh isolation environment. According to the study Li et al. (2014), microbial communities from extreme environments tend to evolve faster than those from less stressful ones. Moreover, the genomes investigation obtained from the metagenomes of Arctic samples (MAGs) showed that genomes exclusively distributed in Arctic regions displayed a notably larger size than those present in lower latitudes. However, there were no significant differences between their coding densities (Royo‐Llonch et al. 2021). In the case of studied genera, the consistent distribution of genes in each COGs category was observed. The observed relations may facilitate an adaptation to a copiotrophic lifestyle in environments with high resource availability (Royo‐Llonch et al. 2021), thus, in the case of bioremediation of contaminated environments, they may potentially enhance the bacteria's ability to inhabit new ecological niches successfully.

**FIGURE 2:**
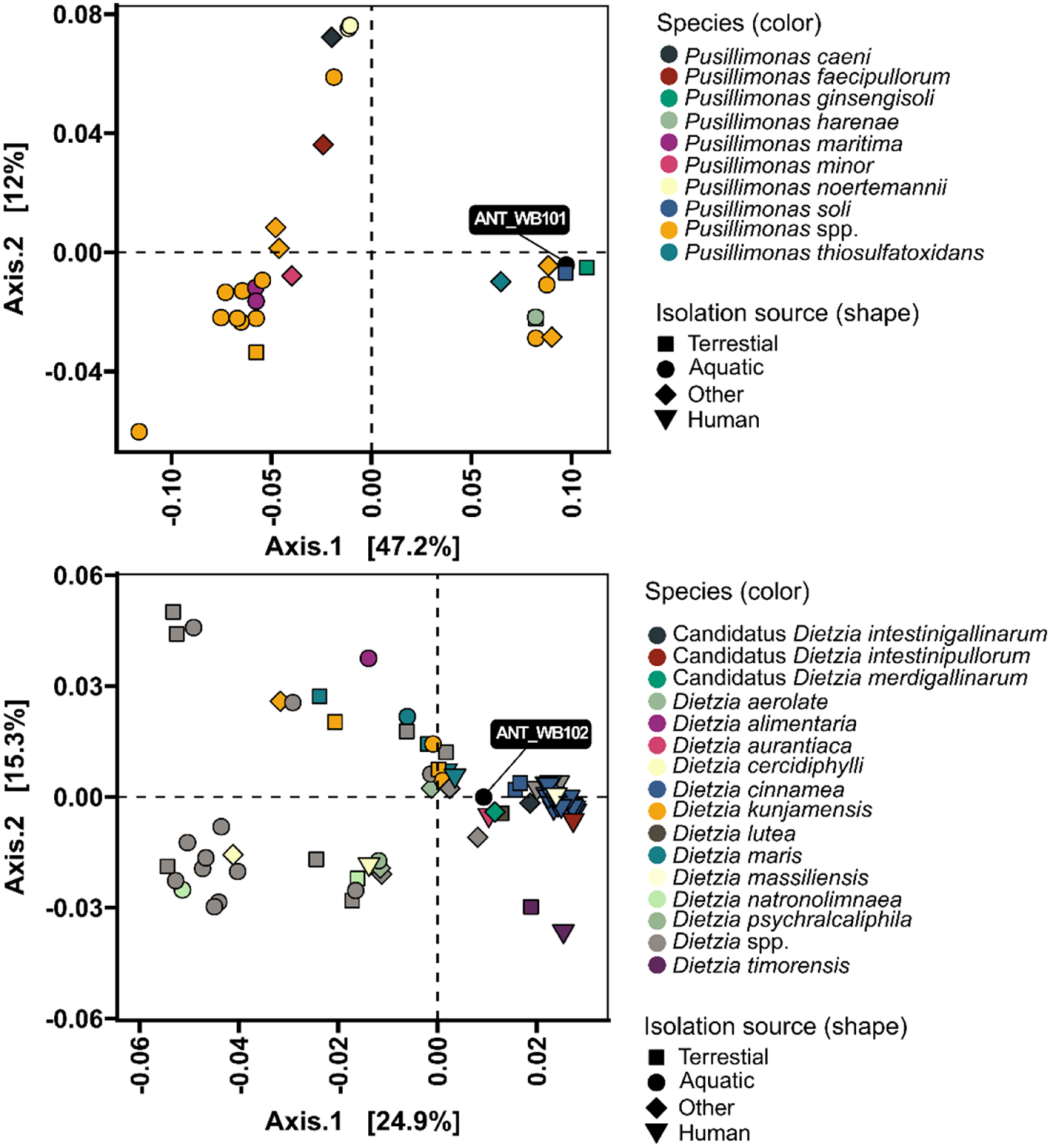
PCoA analysis with JSD similarity matrix based on KEGG KO annotated data of *Pusillimonas* and *Dietzia* genes.

The subsequent comparative analysis based on PEG‐assigned KO numbers (KEGG) and PCoA analysis with the JSD similarity matrix indicated that the first two principal components account for 59.2% and 40.2% of the variability among *Pusillimonas* and *Dietzia* species respectively (Figure 2). In addition, they indicate the high similarity of isolated bacteria to strains obtained from different environments—terrestrial, aquatic, or associated with a symbiosis with humans. Among them, the most similar strains based on the JSD matrix and for additional confirmation the Jaccard matrix are *P*. spp. TS35 (freshwater sediment), DSM 25264 (farm soil), KCTC 22455 (farm soil), KCTC 22046 (farm soil), JCM 16917 (beach sand), DSM 25667 (tidal flat), M17 (topsoil) and T7‐7 (Bohai Sea) and *D*. spp. B32 (mare), DSM 44907 (cold desert), 111 N12‐1 (South China Sea seawater), J3 (landfill leachate), DSM 45139 (surface‐sterilised stem sample), 55 (root nodule), p3‐SID1051 (human), BP‐168 (pistachio trees), p3‐SID1379 (human), p3‐SID1371 (human), W5195 (human) and p3‐SID826 (human).

The data indicates the metabolic similarity of ANT_WB101 with strains obtained from terrestrial environments and found in agricultural soils. However, there is a lack of studies indicating the impact of these bacteria on soil health and plant growth. In the case of ANT_WB102 there was identified metabolic similarity to species such as *D. aurantiaca* , *D. cinnamea* and *D. maris* . These taxa contained bacteria both isolated from environmental sources and human clinical specimens. Studies conducted up to date indicate the pathogenic potential of these species, especially in immunocompromised patients (Brown et al. 2022; Koerner et al. 2009). However, some *Dietzia* strains were found to exhibit plant growth‐promoting properties (Gusain et al. 2016) including *D. cinnamea* 55 (Khan et al. 2020). This strain was also tested for pathogenicity against plants, larvae and nematodes, all of which yielded no adverse effects.

### 3.2 Physiological Characterisation and Attributes Against Expositions on Hydrocarbons and Heavy Metals

The use of bacteria in bioremediation without proper physiological and tolerance analysis may result in ineffective outcomes and may even be environmentally harmful. A thorough understanding of the characteristics of bacteria helps ensure that bioremediation efforts will be effective and environmentally safe. Therefore, we tested the tolerance of the strains to growth under different environmental conditions, resistance to low temperature and tolerance to high hydrocarbon and heavy metal concentrations.

**FIGURE 3:**
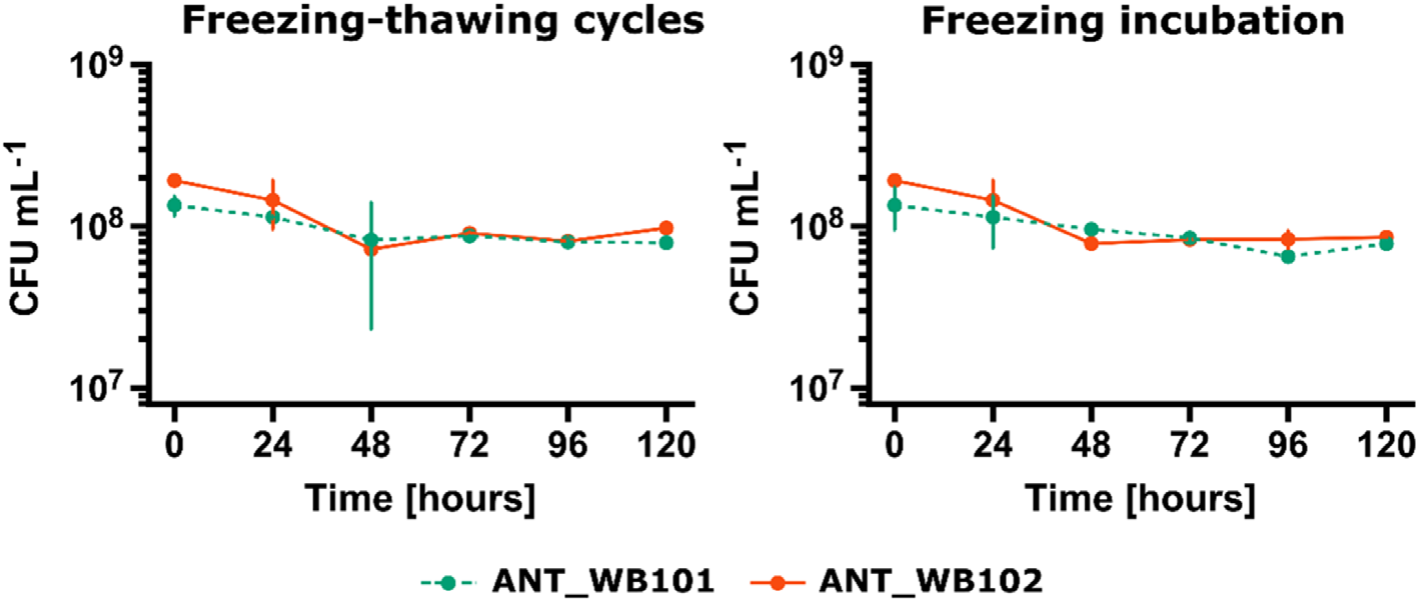
Analysis of tolerance of ANT_WB101 and ANT_WB102 strains: The left graph shows the freeze–thaw cycles with temperature changes from −20°C to +4°C, and the right graph shows the analysis of survivability of bacteria exposed to incubation at −20°C. To the test, bacteria were cultured in a nutrient‐rich medium (LB) at optimal growth temperature and subsequently incubated at +4°C for 24 h to adapt to low temperatures. The data are presented as the mean ± SD of the triplicate experiments.

The results show that the analysed bacteria could grow over a wide temperature range 4°C–37°C, pH 4/5–11 (ANT_WB102/ANT_WB101) and salinity of 0–60/90 g L^−1^ (ANT_WB101/ANT_WB102). Optimum growth for both strains was observed at 22°C–30°C and pH 7–8. Survival analysis after freezing and freeze–thaw cycles showed resistance to negative ambient temperatures, with the decrease of bacterial counts by order of magnitude from 10^8^ to 10^7^ CFU mL^−1^ (Figure 3).

Strains showed the ability to grow in the presence of heavy metals—Co(II) up to a concentration of 0.8 mM (47.2 mg L^−1^) and Mo(II) up to concentrations of 500 (48,000 mg L^−1^) and 50 mM (4800 mg L^−1^) for ANT_WB101 and ANT_WB102, respectively. For Cu(II), Mn(II), Zn(II), and As(V), no growth was observed at concentrations above 0.1 mM (> 5.5 mg L^−1^). These values are notably lower than those observed in oil‐contaminated sites, which could be a limiting factor in the context of bioremediation efforts. However, the distribution of metals in the soil could be inconsistent, and their concentrations varied due to the composition of crude oil, different chemical and oxidation forms (Borah and Deka 2023). Additionally, the strains showed survivability in oil‐contaminated soil at concentrations of 500–20,000 mg kg^−1^.

**FIGURE 4:**
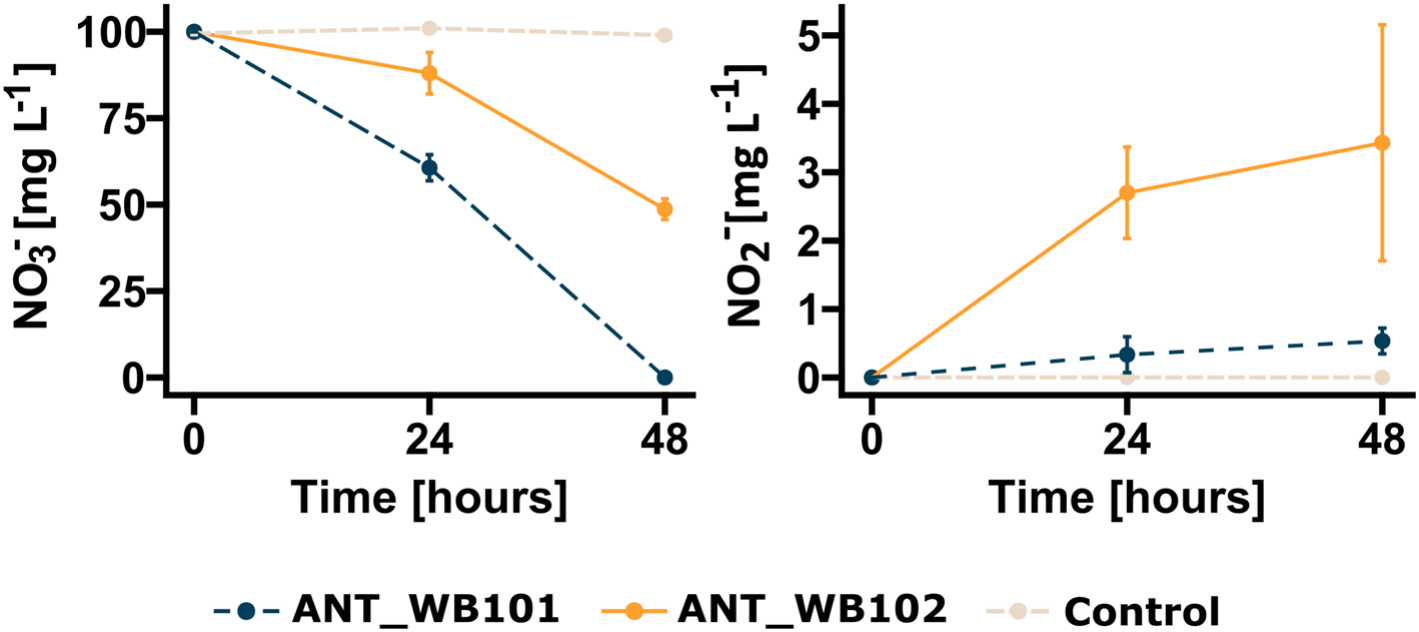
Analysis of nitrate reduction rate (mean ± SD; *n* = 3) of ANT_WB101 and ANT_WB102 in anoxygenic cultures conducted at 22°C under 180 rpm agitation for 48 h. The left graph shows changes in nitrate concentration, and the right graph shows changes in nitrite concentration.

In previous study (Krucon et al. 2021), including tests on denitrification, it was demonstrated that ANT_WB101 could reduce nitrates to atmospheric nitrogen. Subsequent analysis of the genomes of both strains confirmed the presence of denitrification‐related genes in ANT_WB101 and revealed the presence of genes encoding nitrate and nitrite reductase in ANT_WB102 (Table S3). Functional analysis performed at 22°C showed that within 48 h, the ANT_WB101 completely reduced the nitrate content in the medium from 100 mg L^−1^ (with a nitrite content of 0.53 ± 0.19 mg L^−1^). In contrast the ANT_WB102 reduced the nitrate content to 49 ± 3.0 mg L^−1^ (NO_2_^−^—3.43 ± 1.7 mg L^−1^) (Figure 4). These results indicate the potential of these strains in the treatment of low‐oxygen or anoxygenic environments.

Previous research also demonstrated that ANT_WB102 is capable of producing biosurfactants when grown on R2A medium supplemented with vegetable (canola) oil. To assess their potential for producing surface‐active compounds applicable in bioremediation, production tests were conducted using BH minimal medium supplemented with cost‐effective organic resources such as glycerol and molasses. However, after a week of cultivation, no surfactants were detected in any of the tested media.

### 3.3 Biochemical Potential in Bioremediation Based on Genomic Data

In the genomes of both strains, genes encoding enzymes associated with the degradation of complex organic compounds and related to the degradation of (1) alkanes and (2) aromatic compounds were identified. The first group includes alkane monooxygenase (*ladA*/*alkB*) found in both strains. The alkane monooxygenase identified in ANT_WB101 belongs to the category of flavoprotein monooxygenase (Li et al. 2008). This monooxygenase utilises a terminal oxidation pathway for the conversion of long‐chain alkanes up to at least C36 (Feng et al. 2007)—nevertheless, no homologues of the Rieske‐type alkane monooxygenase genes found in *Pusillimonas* sp. T7‐7 (Li et al. 2013) were identified in the ANT_WB101 genome. The monooxygenase identified in ANT_WB102 is a member of alkane hydroxylase, which requires a fused rubredoxin domain to perform its function and enables the degradation of alkanes up to C32 in length (Nie et al. 2011). The second group includes polyphenol oxygenase PPO present in both strains and propane/phenol monooxygenase present in ANT_WB102. Polyphenol oxidase plays a key role in the degradation of aromatic hydrocarbons of phenolic compounds into humus in soil. This enzyme is an important indicator of microbial activity, especially in contaminated soils (Gianfreda et al. 2005). In both strains, the identified genes were annotated as peptidoglycan editing factor PgeF containing copper oxidase (laccase) domain. The research conducted on the homologous protein (CAK32504.1) showed its versatile capacity to oxidise a wide range of substrates including syringaldazine, 2,6‐dimethoxyphenol, veratryl alcohol, guaiacol or 4‐methoxybenzyl alcohol (Beloqui et al. 2006). Propane/phenol monooxygenase represents a multicomponent enzyme system, belonging to the propane/methane/phenol/toluene hydroxylase family (IPR003430). Four genes capable of forming the common operon methane monooxygenase (*prmA*), 2Fe‐2S iron–sulphur cluster binding domain‐containing protein (*prmB*), aromatic/alkene monooxygenase hydroxylase subunit beta (*prmC*), MmoB/DmpM family protein (*prmD*), amidohydrolase, iron–sulphur cluster assembly protein and NAD(P)‐dependent alcohol dehydrogenase (*prmE*) and chaperonin GroEL (groL1) were identified, which also correspond in alignment to genes identified in *Rhodococcus* sp. RHA1. The studies (Sharp et al. 2007) showed that this strain could cometabolically degrade N‐nitrosodimethylamine using propane monooxygenase and its activity against this compound increases during growth on propane. Additional analysis of the protein sequences encoded by the *prmA* and *prmC* genes of ANT_WB102 revealed the presence of domains associated with the phenol and its methylated derivatives metabolism to catechol (Nordlund et al. 1990).

In addition, in both genomes, genes encoding catalase and cytochrome P450 were identified. In the ANT_WB102, chloride peroxidase‐enzymes were also identified which can non‐specifically oxidise organic compounds and dye‐decolourising peroxidase. The ANT_WB101 contains genes related to dehalogenation as well: 2‐haloacid dehalogenase and haloalkane dehalogenase. In the genome of ANT_102, the genes encoding cytochrome P450 belong to the CYP153 family (CYP153) (Funhoff et al. 2006) and out of the nine identified genes as P450, six are part of this family. Their function is the hydroxylation of medium‐chain‐length alkanes. Analysis of CYP153 identified in *Dietzia* sp. DQ12‐45‐1b revealed its role in hydroxylating n‐alkanes shorter than C10, while research indicated that AlkB‐like alkane hydroxylases was responsible for acting within those longer than C14 (Nie et al. 2014). In the case of ANT_WB101, two P450‐encoding genes were identified. One of these genes, containing a CYP130‐like region, that may be associated with binding primary arylamines (Podust et al. 2009) and PDR_like phthalate dioxygenase reductase was also noted. These findings underscore the variety of enzymes evolved in pristine environment in the analysed strains with the potential to treat crude oil pollution. The information about the above genes is summarised in Table S3.

### 3.4 Genomic Analysis of the Strains in Terms of Biosafety and Functional Analysis of Antibiotic Susceptibility

The introduction of new bacteria into the environment should not lead to harmful interactions with humans, animals, or plants. Due to the close affinity of ANT_WB102 with bacteria identified as opportunistic pathogens, and the occurrence of pathogenic or opportunistic pathogenic bacteria such as *Bordetella* (Bridel et al. 2022), *Achromobacter* (Veschetti et al. 2022) and *Alcaligenes* (Huang 2020) within the Alcaligenaceae family, comprehensive genomic analysis was undertaken to identify potential virulence factors in the studied strains.

Resistome analysis based on RGI protein sequence homology showed that the analysed strains may exhibit the following resistance mechanisms: (1) antibiotic efflux; (2) antibiotic target alteration; (3) antibiotic target protection; (4) antibiotic target replacement; (5) antibiotic inactivation and (6) reduced permeability to antibiotics (Table S4). However, the matches identified as strict include genes encoding proteins related to the antibiotic efflux resistance mechanism: small multidrug resistance (SMR) antibiotic and resistance‐nodulation‐cell division (RND) antibiotic efflux pump in the case of ANT_WB101. These genes are involved in resistance to disinfectants, antiseptics, fluoroquinolone antibiotics and tetracycline antibiotics. In the case of ANT_WB102, the gene encoding aminosalicylate‐resistant dihydrofolate synthase and antibiotic target alteration was found.

The antibiotic resistance analysis revealed that each of the tested compounds causes bacterial growth inhibition (Table 1). There are no appropriate guidelines for *Pusillimonas* and *Dietzia* to assign them as susceptible or resistant bacteria. Previous studies of susceptibilities of *Dietzia* isolates revealed their antibiotic‐sensitivity to amikacin, amoxicillin‐clavulanic acid, ampicillin, ceftriaxone, ciprofloxacin, clarithromycin, trimethoprim‐sulfamethoxazole, imipenem, linezolid, minocycline, tobramycin, and vancomycin (Pilares et al. 2010). Moreover, a study involving *D. maris* indicated it sensitive to aztreonam, ciprofloxacin, meslocillin, oxacillin, penicillin G, perfloxacin, and ticarcillin and demonstrating resistance to sulfamethoxazole in the disk diffusion test (Bizet et al. 1997).

**TABLE 1:** Comparison of the antibiotic resistance profiles of ANT_WB101 and ANT_WB102 strains.

Group	Antibiotic (disc load [μg])	Inhibitory zone (mm)	Susceptibility
*Pusillimonas* sp. ANT_WB101			
Aminoglycosides	Gentamicin (10)	36	S^a^
	Neomycin (30)	36	S^a^
Carbapenems	Imipenem (10)	37	S^a^
	Meropenem (10)	33	S^a^, ^b^
Cephalosporins	Cefepime (30)	14	R^a^
	Cefuroxime (30)	12	R^a^
Fluoroquinolones	Ciprofloxacin (5)	22	S^a^
Monobactams	Aztreonam (30)	36	S^a^
Penicillins	Amoxicillin/clavulanic acid (30)	17	I^a^
	Ampicillin (10)	18	I^a^
	Piperacillin/tazobactam (36)	22	I^a^
Polymyxin	Colistin (10)	22	R^b^/S^a^
Miscellaneous	Fosfomycin (50)	36	S^a^
	Trimethoprim (5)	22	S^a^
	Trimethoprim/sulfamethoxazole (25)	22	R^b^/S^a^
*Dietzia* sp. ANT_WB102			
Aminoglycosides	Gentamicin (10)	37	S^a^, ^c^
	Streptomycin (25)	37	S^a^, ^c^
Cephalosporins	Cefotaxime (5)	36	S^a^, ^c^
	Ceftriaxone (30)	37	S^a^, ^c^
Glycopeptides	Teicoplanin (30)	30	S^a^
	Vancomycin (5)	36	S^a^, ^c^
Linkosamides	Clindamycin (2)	37	S^a^
Macrolides	Erythromycin (15)	37	S^a^
Oxazolidinones	Linezolid (10)	34	S^a^, ^c^
Penicillins	Ampicillin (10)	18	I^a^, ^c^
Tetracyclines	Tetracycline (30)	33	S^a^
	Tigecycline (15)	37	S^a^
Miscellaneous	Chloramphenicol (30)	18	S^a^, ^c^
	Trimethoprim (5)	28	S^a^, ^c^

Abbreviations: I, intermediate; R, resistant; S, sensitive.

^a^ Carret et al. (2003).

^b^ According to *Achromobacter* (The European Committee on Antimicrobial Susceptibility Testing 2023. Breakpoint tables for interpretation of MICs and zone diameters. Version 13.1).

^c^ According to *Nocardia* (Lebeaux et al. 2019; Wallace and Steele 1988).

Comparing the genomes of the two strains with the VFs database revealed factors associated with (1) adherence, (2) biofilm formation, (3) effector delivery system, (4) exotoxin, (5) immune modulation, (6) motility, (7) nutritional/metabolic factor, (8) regulation and (9) stress survival (Table S5). The first group in ANT_WB101 includes proteins associated with curli fibres extracellular fibres promoting biofilm formation in the extracellular matrix. In both strains, chaperonin GroEL which mediate a complement‐independent attachment to host cells, and the elongation factor Tu mediates attachment by interacting with host cell nucleolins were identified as well. Determinants associated with biofilm production identified in the ANT_WB101 genome include PNAG biosynthesis, a molecular chaperone potentially associated with a role in the initial steps of biofilm formation, and transporters that play roles in the transport of autoinducer molecules during biofilm formation. In addition, genes related to the I, II, III and IV secretion system were identified in these bacteria. A phospholipase D was identified as an exotoxin in both strains, with a role in pathogenesis related to the phospholipids cleavage present in the host cell membrane, as well as the phospholipids degradation located on mucosal barriers to facilitate bacterial invasion. Group 5 includes factors responsible for the formation of capsules, lipopolysaccharides (ANT_WB101) or lipoarabinomannan (ANT_WB102), and outer membrane protein A (OmpA) in ANT_WB101, which, along with porin and C40 family peptidase, may also be associated with invasion. Genes related to chemotaxis, such as flagellar motor switch adaptation and twitching mobility, are responsible for motility in ANT_WB101. Nutritional/metabolic factors are related to nitrate respiration: nitrate reductase, biotin synthesis, siderophores and their transport and heme transport and degradation (hemolysin protein as an exoenzyme), aspartate 1‐decarboxylase (*panD*) and pantoate biosynthesis. The found regulators include response regulator, rensponse regulator transcription factor, sigma‐70 family RNA polymerase sigma factor and two component systems MtrAB and KdpE. The last group includes superoxide dismutase, peroxiredoxin, catalase, ATP‐dependent chaperone ClpB, ATP‐dependent protease ClpA, ATP‐dependent Clp endopeptidase and ATP binding cassette (ABC) permease (ANT_WB101). Furthermore, among the identified potential virulence factors were isocitrate lyase (*icl*) and the signal recognition particle receptor FtsY. Notably, for the strain ANT_WB102, a selection of genes including *coaA*, *ftsY*, *mprA* and *sigH*, as well as *mtrA* and *icl*, were recognised as part of the core genes within the *Dietzia* pangenome (dos Santos et al. 2023).

### 3.5 Degradation of Hydrocarbons in Liquid Cultures

To test the ability in breaking down aliphatic and aromatic hydrocarbons, bacterial activity was evaluated under both aerobic and anaerobic conditions, with crude oil as the only source of carbon and energy. Both strains showed a decrease in the hydrocarbons content after 14 days of cultivation. The greatest decrease occurred under aerobic conditions in the case of ANT_WB102: to 0.51 ± 0.16 g L^−1^ with 8.00 ± 0.12 g L^−1^ in the control variant (94% efficiency). ANT_WB102 achieved a decrease to 1.66 ± 0.41 g L^−1^ (79% efficiency). As for the anaerobic conditions, where nitrate was chosen as the electron acceptor, a reduction of hydrocarbons was achieved to 3.68 ± 0.30 g L^−1^ for ANT_WB101 and 5.83 ± 0.47 g L^−1^ for ANT_WB102 (42% and 34% efficiency, respectively). The achieved degradation efficiency of ANT_WB102 is similar to the results obtained in previous studies, which focused on the degradation potential of *Dietzia* strains isolated from hydrocarbon‐contaminated environments. For instance, in the study examining the activity of *D. maris* AURCCBT01X, a degradation efficiency of 91.87% (with 0.75% w v^−1^ crude oil) was achieved within 7 days (Venil et al. 2021). Another study involving *D. cinnamea* KA1 showed a degradation efficiency of 95.7% (1.00% v v^−1^ crude oil) within 5 days (Kavynifard et al. 2016), and in the case of *D*. sp. CN3, degradation was at 91.6% (with 0.5% w v^−1^ crude oil) achieved in 12 days (Chen et al. 2017).

### 3.6 Degradation of Hydrocarbons in Contaminated Soil

The applications of microorganisms in environmental contexts require comprehensive tests, including examinations of their ability to degrade contaminants under conditions reflective of relevant or operational environments. These strains should indicate cost‐effectiveness, effectively leading to increased pollutant degradation efficiency. Therefore, functional assessments of hydrocarbon compounds' breakdown were coupled with studies of the efficacy of strains performed in environmental samples.

To evaluate the effect of the strains on the degradation process, the soil contaminated with petroleum hydrocarbons was treated with these bacteria and a medium containing N–NH4, N–NO3 and a phosphorous source. This soil (clayey silt) exhibited an imbalanced C:N ratio of 16:1, with a pH of 6.68. The potential for bioremediation was evaluated at both 22°C and 4°C, representing different seasonal conditions. The initial bacterial count in untreated soil was 2.81 ± 0.55 × 10^5^ CFU g^−1^ DW and counts were 2.80 ± 0.48 × 10^6^ CFU g^−1^ DW for ANT_WB101 treated soil and in the case of ANT_WB102 2.10 ± 1.5 × 10^6^ CFU g^−1^ DW. After 28 days of incubation in the OxiTop system, the abundance of bacteria exposed to 4°C remained relatively constant and amounted to 7.22 ± 2.38 × 10^5^, 1.99 ± 0.59 × 10^6^ and 2.06 ± 0.10 × 10^6^ CFU g^−1^ of dry soil in the control variant, ANT_WB101 and ANT_WB102, respectively. At 22°C for these variants, counts increased by an order of magnitude to 4.21 ± 1.44 × 10^7^, 2.18 ± 0.87 × 10^7^ and 2.25 ± 0.24 × 10^7^ CFU g^−1^ of dry soil.

**FIGURE 5:**
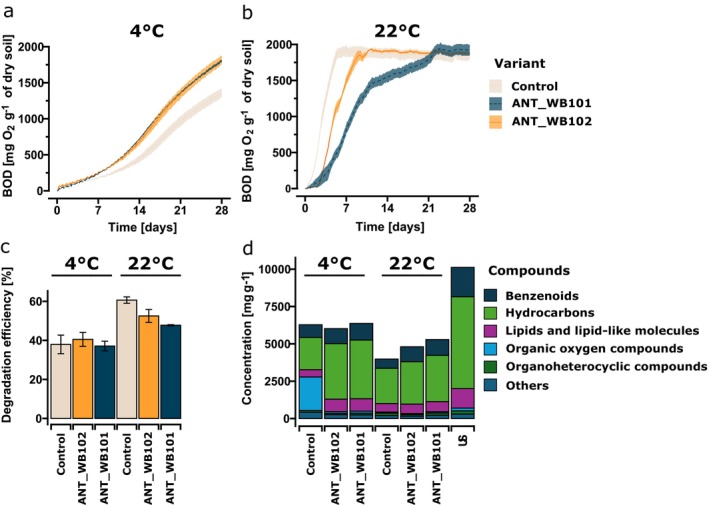
Comparison of Biological Oxygen Demand (BOD) of hydrocarbon‐contaminated soil inoculated with *Pusillimonas* sp. ANT_WB101 or *Dietzia* sp. ANT_WB102 and appropriate control variants (a, b). Abbreviation: US, untreated soil. In all cases, the soil was treated with Bushnell‐Haas medium as well. Summary of degradation efficiency of hydrocarbon pollution from the contaminated soil based on the activity of the indigenous community (presented in all cases) and the activity of the seeded bacteria ANT_WB101 and ANT_WB102 (c, d). The data are expressed as the mean ± standard deviation (SD) from duplicate experiments with three technical replicates each.

Microorganism activity was assessed based on the values of biological oxygen demand (BOD) and hydrocarbon content (Figure 5). The highest increase in BOD was observed at 22°C in the case of soil stimulated with native microorganisms during the first week of incubation and reached a plateau on the fifth day at 1.87 g O_2_ g^−1^ of dry soil. In the case of bioaugmentation with the *Dietzia* the plateau occurred on the ninth day and in the case of the *Pusillimonas* on the 21st day. At a lower ambient temperature, the highest BOD value of 1.87 g O_2_ g^−1^ DW was achieved for ANT_WB101 and ANT_WB102, compared to the control variant with a BOD value of 1.4 g O_2_ g^−1^ of dry soil.

**FIGURE 6:**
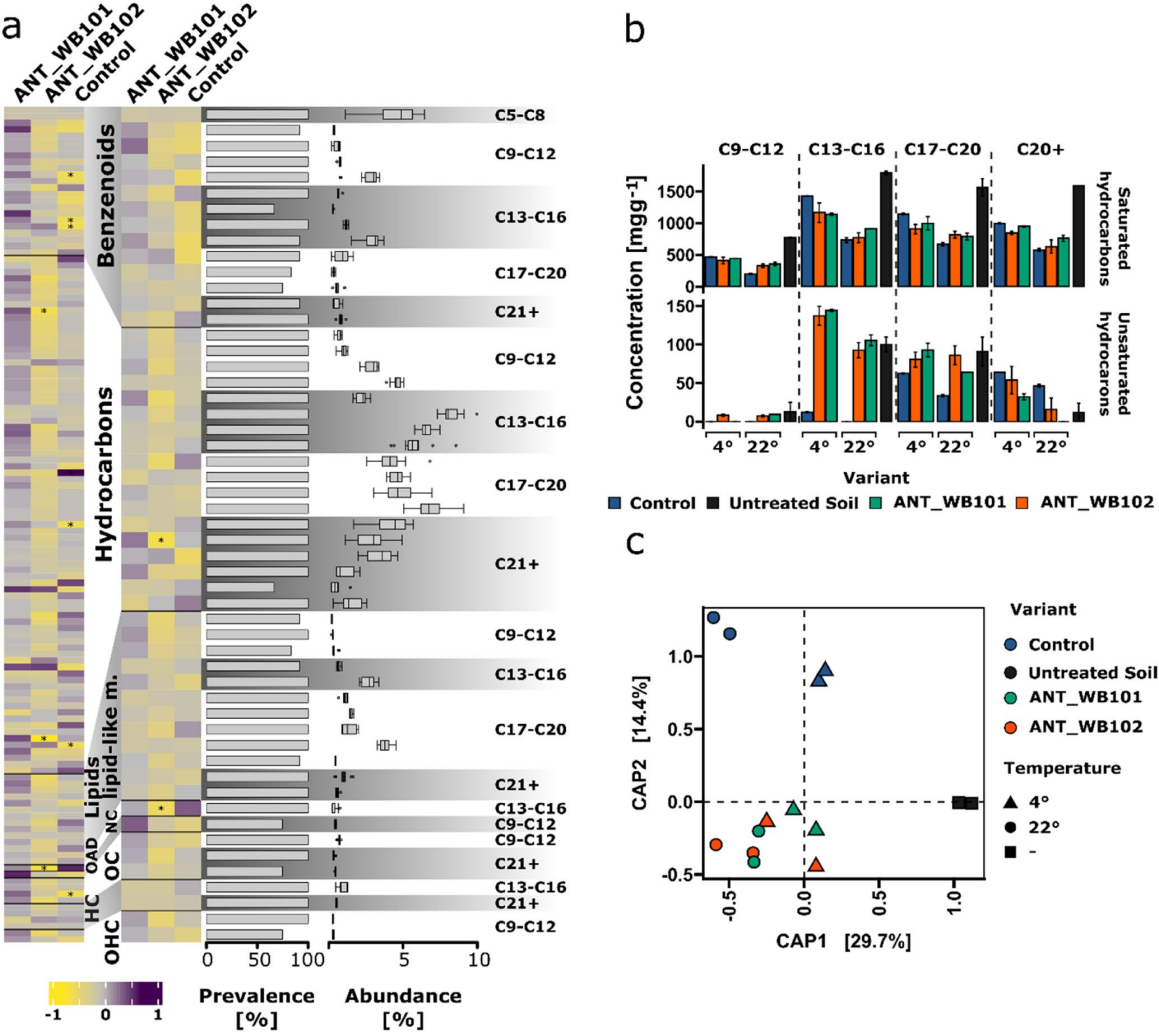
Relationship analysis between the organic compounds' content within the treated soil and the applied bioremediation treatments. (a) Evaluation of correlations between bioagumentation and biostimulation variants and individual compounds and compound groups; (b) comparison of the saturated hydrocarbons content (upper plot) and unsaturated hydrocarbons (lower plot) of different molecular lengths; (c) similarity examination between the studied samples based on their organic compound content. In the (a) the colour spectrum ranging from yellow to purple signifies the correlation strength between the analysed compound content in the samples. The yellow colour indicates lower content, whereas purple indicates higher content. Abbreviations: HC, organohalogen compound; NC, nucleosides, nucleotides and analogues; OAD, organic acids and derivatives; OC, organic oxygen compound; OHC, organoheterocyclic compounds. The data are expressed as the mean ± standard deviation (SD) from duplicate experiments with three technical replicates each.

Evaluation of organic compound content through GC–MS analysis revealed varying degradation efficiencies (Figure 5c). The highest degradation efficiency was observed at 22°C: 53.64% ± 1.24% in contrast to 38.51% ± 0.93% at 4°C. Among the tested conditions, the highest efficiency showed biostimulated native microorganisms: 60.64% ± 1.64%. Following were the samples with *Dietzia*—52.53% ± 3.31% and *Pusillimonas* with 47.76% ± 0.28% degradation efficiency. Under the 4°C incubation conditions variants demonstrated comparable efficiencies of 40.51% ± 3.59%, 37.10% ± 2.47% and 37%.94% ± 4.76% for the *Dietzia*‐inoculated, *Pusillimonas*‐inoculated, and uninoculated control samples, respectively. The compounds identified through untargeted chromatographic analysis were classified based on the ClassyFire database. Specific groups including benzenoids, hydrocarbons, lipids and lipid‐like molecules, organic oxygen and organoheterocyclic compounds were chosen for comparison (Figure 5d). The control variants showed a 2.25‐fold decrease in benzenoid compounds, 2.72‐fold of hydrocarbons and 2.47‐fold of content lipids and lipid‐like molecules in comparison to untreated soil. In the case of seeding with Antarctic bacteria, there was a reduction by a factor of 1.88, 1.84 and 1.77‐fold for the respective groups compared to the initial conditions. Organic oxygen compounds and organic heterocyclic compounds exhibit a 2.11‐fold and 1.96‐fold decrease in the inoculated soil, respectively, while in the biostimulated soil these compounds decreased by 1.49‐fold and 1.62‐fold for the first and second groups, respectively. To examine the influence of the applied bacteria and the medium on the degradation of specific compounds and compound groups, correlation analyses were performed with a 60% prevalence threshold (Figure 6a). The analysis revealed that a statistically significant correlation related to the decrease in contamination within compound groups (correlation coefficient *r* > 0.5, q‐value < 0.05) occurred only with *Dietzia*‐inoculated soil for C21+ hydrocarbons (docosane and apart from groups 5‐methyl decane) and nucleosides, nucleotides and analogues (2‐fluoro‐1‐triacetylribofuranosyl). Whereas, within control variants significant correlation was observed only within individual compounds—benzenoids: 2.3‐dimethyl naphthalene, pyrene and triphenylene, hydrocarbons: 3‐methyl tridecane, heneicosylcyclopentane and organic oxygen compounds: [(1R.3S)‐3‐(aminomethyl)‐1.2.2‐trimethyl‐cyclopentyl]methanol. However, it is crucial to consider groups of compounds, as isomeric compounds even with high similarity of match could be misclassified.

Variance analysis and subsequent post hoc tests performed for hydrocarbon groups showed statistically greater, significant differences in degradation observed in variants inoculated with bacteria, but exclusively at 4°C (Figure 6b). Specifically, it related to saturated hydrocarbons C20+ in the case of ANT_WB102 and saturated hydrocarbons C9–C16 in the case of ANT_WB101. The overall correlation pattern between the variants and the compounds indicates a distinct impact of the applied conditions on the degradation processes. Nevertheless, the samples' comparative analysis indicated a greater similarity between the samples with the applied bacteria as opposed to the case only of biostimulation (Figure 6c).

The results revealed that bioaugmentation with Antarctic bacteria and biostimulated indigenous microbial communities exhibited considerable results in the degradation of contaminants in the soil. Variations in BOD reaching a plateau indicated that the introduction of additional microorganisms influenced native microbial communities and altered the soil's metabolic profile. Especially in colder environments, enhancing metabolic activity is crucial for expediting long‐term cleanup processes at contaminated sites. At 22°C, the slower increase in BOD values in bioaugmented soil may be attributed to the strong competition from indigenous microorganisms, resulting in the extended time required for the introduced bacteria to adapt and establish a microbial community. This could also explain why the application of these strains did not lead to significant enhancements in biodegradation, despite the high potential for contaminant breakdown based on genomic analyses.

Previous studies suggest that biostimulation can effectively enhance the remediation of contaminated soils (Banet et al. 2021; Wu et al. 2016; Abed et al. 2015; Sayara et al. 2011), by increasing the population of degraders—a key factor for enhancing treatment efficiency (Wu et al. 2016). However, in harsh soil conditions, biostimulation alone may be insufficient. In such cases, introducing bacteria highly adapted to specific contaminants can accelerate treatment processes (Al‐Kaabi et al. 2018). These findings could be attributed to the behavior of individual isolates or consortia, which can vary in response to contaminant concentrations. Strains that may be sensitive to the toxicity of total petroleum hydrocarbons (TPH), the toxic effects of metabolites, or end‐product toxicity, as well as substrate depletion, could exhibit better tolerance in the presence of other bacteria (Alsayegh et al. 2021). Therefore, successful remediation of contaminated sites relies on precise environmental assessment, the selection of appropriate microbial strains and the monitoring of the selected strategy.

## 4 Conclusions

This study showed that psychrorolerant strains, *Pusillimonas* sp. ANT_WB101 and *Dietzia* sp. ANT_WB102, isolated from an uncontaminated environment, possess valuable traits for bioremediation processes. They demonstrate metabolic versatility, crucial for effective crude oil degradation in aerobic and anaerobic conditions. Their wide‐range tolerance to various environmental factors, enables them to thrive in challenging environments, including those exposed to freezing–thawing cycles. Although the genomes of these bacteria revealed potential virulence factors and resistance genes commonly found in environmental bacteria, they showed sensitivity to several classes of antibiotics. This indicates a low risk of pathogenicity, supporting their safety for environmental applications. However, their effectiveness in treating oil‐contaminated soil in lab‐scale bioremediation was comparable to the activity of biostimulated native microorganisms, underlining the potential challenges of using microorganisms not adapted initially to the site‐specific conditions. Adaptation to adverse environments with competition and integration with indigenous microbes to establish effectiveness in the community could be associated with extended bioremediation time and requires continuous monitoring. Nonetheless, the observed increase in soil respiration at lower ambient temperatures after bioaugmentation with Antarctic strains highlights their promise for hydrocarbon degradation in cold environments. The comparative analysis with other bacterial genomes revealed their distinct genetic traits. This indicates that even unpolluted environments such as polar ones, could offer promising bioremediation prospects due to the rigorous conditions promoting adaptation without reducing the versatility of these microorganisms. Overall, our study indicates the great potential of novel *Pusillimonas* and *Dietzia* isolates in environmental applications.

## Author Contributions

**Tomasz Krucoń:** conceptualization, methodology, investigation, visualization, data curation, validation, writing – original draft preparation, writing – review and editing. **Julia Karbowska:** investigation. **Wiktoria Pietrowicz:** investigation. **Robert Stasiuk:** investigation. **Łukasz Drewniak:** supervision, writing – review and editing, funding acquisition.

## Funding

This study was funded by the Foundation for Polish Science (grant no. POIR.04.04.00‐00‐14E6/18).

## Conflicts of Interest

The authors declare no conflicts of interest.

## Supporting information


**Data S1:** Genetic information related to the ability of bioremediation potential, biosafety and comparison to other genomes of *Pusillimonas* and *Dietzia* strains.

## Data Availability

The data that support the findings of this study are available on request from the corresponding author. The data are not publicly available due to privacy or ethical restrictions.
