# Supplementary material for: Exploring the Potential and Evaluating Hydrocarbon Degradation by Novel Antarctic Dietzia and Pusillimonas Isolates From a Pristine Environment
Source: Environ Microbiol Rep. 2026 Jan 8;18(1):e70248. doi: 10.1111/1758-2229.70248 (PMC12783916; doi:10.1111/1758-2229.70248)
Supplement: Supplementary file 1 — Data S1: Genetic information related to the ability of bioremediation potential, biosafety and comparison to other genomes of Pusillimonas and Dietzia strains. [file EMI4-18-e70248-s001.docx]

Supporting Information

Exploring the potential and evaluating hydrocarbon degradation by novel Antarctic *Dietzia*
and *Pusillimonas* isolates from a pristine environment

Tomasz Krucoń^1^, Julia Karbowska^1^, Wiktoria Pietrowicz^1^, Robert Stasiuk^2^, Łukasz Drewniak^1,3*^

^1^ Department of Environmental Microbiology and Biotechnology, Institute of Microbiology,
Faculty of Biology, University of Warsaw, Warsaw, Poland

^2^ Department of Geomicrobiology, Institute of Microbiology, Faculty of Biology, University of Warsaw, Warsaw, Poland

^3^ Department of Microbiology, Institute of Biology, Faculty of Natural Sciences, Jan Kochanowski 9 University, 25-406 Kielce, Poland

*Correspondence: Łukasz Drewniak l.drewniak2@uw.edu.pl, Institute of Microbiology,
Faculty of Biology, University of Warsaw, Ilji Miecznikowa 1, 02-096, Warsaw, Poland

Contents

[1. Comparative and phylogenomic analyses 2](#_Toc168824448)

[Table S1. Genome information of *Pusillimonas* sp. ANT_WB101 and *Dietzia* sp. ANT_WB102. 3](#_Toc168824451)

[Table S2. The most similar strain to ANT_WB101 and ANT_WB102 based on 16S rDNA sequence comparison 4](#_Toc168824452)

[Table S3. Summarize of genes of ANT_WB101 and ANT_WB102 important in bioremediation perspectives. 6](#_Toc168824453)

[Table S4. The results of analysis of resistome of ANT_WB101 and ANT_WB102 using RGI Database. 9](#_Toc168824454)

[Table S5. The results of analysis of virulence factors ANT_WB101 and ANT_WB102 using VFDB. 22](#_Toc168824455)

# 1. Comparative and phylogenomic analyses

## 1.1. Comparative analysis of the genomes of *Pusillimonas* ANT_WB101 and *Dietzia* ANT_WB102 with the genomes of closely related strains


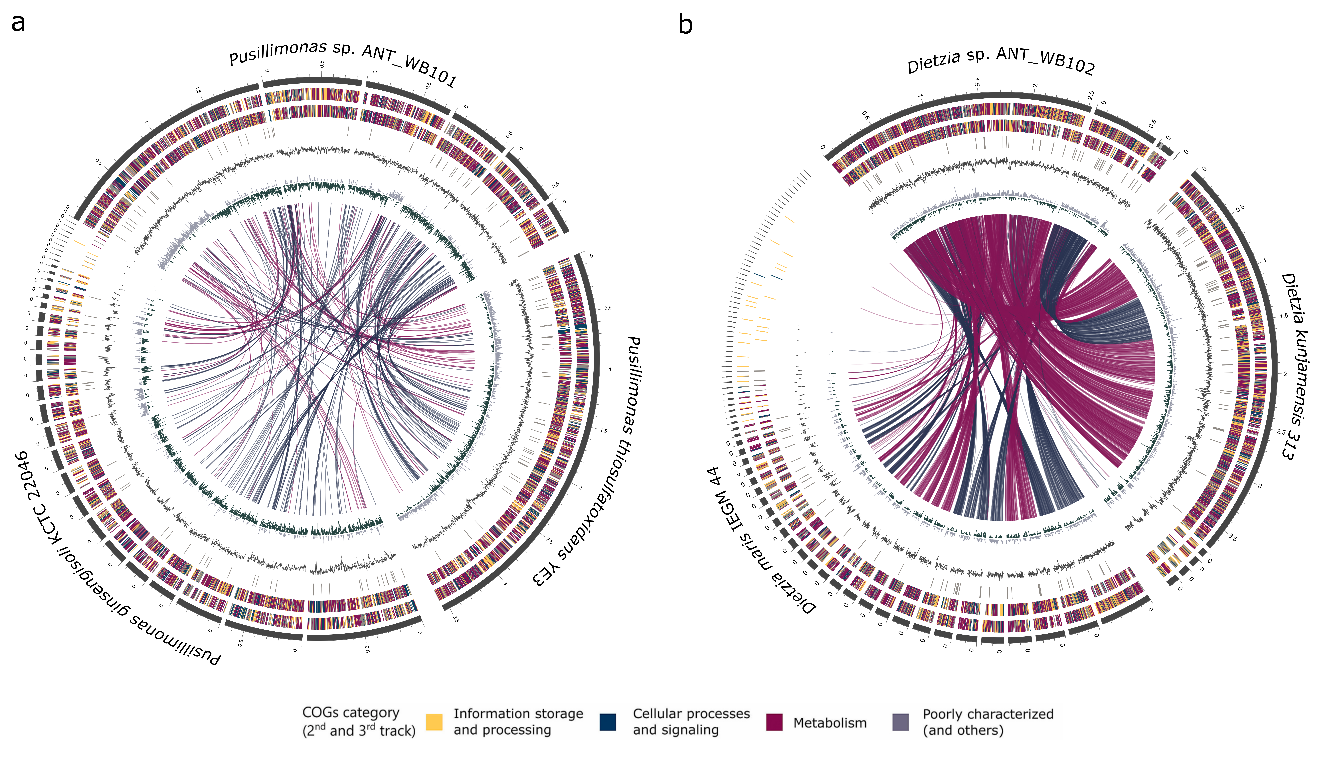


Figure S1. Comparison of the ANT_WB101 and ANT_WB102 with the closely related bacteria of *Pusillimonas* and *Dietzia* genus. The rings shown represent (from the outer): 1) contigs of the genome 2) and 3) ORFs colored according to COG categories located on the forward and reverse strands, respectively; 4) GC-content, 5) GC-skew, and 6) homologous regions occurring between the above genomes – the gray and red colors indicate the same and reverse orientation, respectively. Plots were made using R (v.4.1.0) and Circular Visualization - circlize (v.0.4.13).

## 1.2. Phylogenomic analysis of the genomes of *Pusillimonas* ANT_WB101 and *Dietzia* ANT_WB102 with the genomes within the same genera

Evolutionary histories were inferred by using the Maximum Likelihood method and General Time Reversible model. The trees with the highest log likelihood (-284991.63 and -1025744.3 in the case of *Pusillimonas* and *Dietzia* strains, respectively) are shown. The percentage of trees in which
the associated taxa clustered together is shown next to the branches. Initial tree(s) for the heuristic search were obtained automatically by applying Neighbor-Join and BioNJ algorithms to a matrix of pairwise distances estimated using the Maximum Composite Likelihood (MCL) approach, and then selecting the topology with superior log likelihood value. A discrete Gamma distribution was used to model evolutionary rate differences among sites (5 categories (+G, parameter = 0.4847/0.4336 for *Pusillimonas/Dietzia*)). The rate variation model allowed for some sites to be evolutionarily invariable ([+I], 27.83/27.98% sites). These analyses involved 31 and 80 nucleotide sequences of *Pusillimonas*
and *Dietzia* strains respectively. There was a total of 32137 positions in the first final dataset and 119079 positions in the second dataset.

# Table S1. Genome information of *Pusillimonas* ANT_WB101 and *Dietzia* ANT_WB102.

| Organism name | *Pusillimonas* ANT_WB101 (b-proteobacteria) | *Dietzia* ANT_WB102 (high G+C Gram-positive bacteria) |
| --- | --- | --- |
| BioSample | SAMN12305673 | SAMN12305671 |
| BioProject | PRJNA521297 | |
| GenBank assembly accession | GCA_008369125.1 | GCA_008369165.1 |
| RefSeq assembly accession | GCF_008369125.1 | GCF_008369165.1 |
| Assembly method | SPAdes v. 3.11.1 |  |
| Genome coverage | 80x | 190x |
| Sequencing technology | Illumina MiSeq |  |
| Total sequence length | 4,912,968 | 3,276,615 |
| Total ungapped length | 4,912,968 | 3,276,615 |
| Number of contigs | 6 | 4 |
| Contig N50 | 842,826 | 2,527,158 |
| Contig L50 | 2 | 1 |
| Total number of chromosomes and plasmids | 0 | 0 |
| Number of component sequences (WGS or clone) | 6 | 4 |
| Best-matching type-strain assembly | *Pusillimonas harenae* (GCA_004153685.1) | *Dietzia kunjamensis* (GCA_014144975.1) |
| GC Content | 54.8 | 68.2 |
| WGS Project | VOAY01 | VOBA01 |
| Annotation Pipeline | NCBI Prokaryotic Genome Annotation Pipeline (PGAP) | |
| Genes (total) | 4,438 | 3,054 |
| CDSs (total) | 4,387 | 3,000 |
| Genes (coding) | 4,281 | 2,954 |
| CDSs (with protein) | 4,281 | 2,954 |
| Genes (RNA) | 51 | 54 |
| tRNAs | 45 | 48 |
| ncRNAs | 4 | 3 |
| Pseudo Genes (total) | 106 | 46 |

Table S2. The most similar strain to ANT_WB101 and ANT_WB102 based on 16S rDNA sequence comparison.

| Description | Scientific name | Max score | Total score | Query cover [%] | E-value | Per. ident | Acc. length | Accession |
| --- | --- | --- | --- | --- | --- | --- | --- | --- |
| Query: *Pusillimonas* ANT_WB101 | | | | | | | | |
| *Pusillimonas* sp. strain 2083 16S ribosomal RNA gene, partial sequence | *Pusillimonas* sp. | 2848 | 2848 | 100 | 0.0 | 100.00 | 1542 | MT585892.1 |
| Uncultured bacterium clone UPB-11 16S ribosomal RNA gene, partial sequence | uncultured bacterium | 2795 | 2795 | 99 | 0.0 | 99.61 | 1532 | JX534196.1 |
| *Pusillimonas* sp. T7-7, complete genome | *Pusillimonas* sp. T7-7 | 2712 | 5424 | 100 | 0.0 | 98.38 | 3883605 | CP002663.1 |
| *Pusillimonas thiosulfatoxidans* strain YE3 chromosome, complete genome | *Pusillimonas thiosulfatoxidans* | 2700 | 5394 | 99 | 0.0 | 98.38 | 3548703 | CP022987.1 |
| Alcaligenaceae bacterium strain CGII-56 16S ribosomal RNA gene, partial sequence | Alcaligenaceae bacterium | 2699 | 2699 | 94 | 0.0 | 100.00 | 1461 | MH553025.1 |
| *Pusillimonas thiosulfatoxidans* strain YE3 16S ribosomal RNA, complete sequence | *Pusillimonas thiosulfatoxidans* | 2697 | 2697 | 99 | 0.0 | 98.37 | 1533 | NR_171398.1 |
| *Pusillimonas thiosulfatoxidans* strain YE3 16S ribosomal RNA, complete sequence | *Pusillimonas thiosulfatoxidans* | 2689 | 2689 | 99 | 0.0 | 98.31 | 1532 | NR_171397.1 |
| *Pusillimonas thiosulfatoxidans* strain YE3 16S ribosomal RNA, partial sequence | *Pusillimonas thiosulfatoxidans* | 2669 | 2669 | 99 | 0.0 | 98.30 | 1520 | NR_171479.1 |
| Uncultured bacterium clone 80 16S ribosomal RNA gene, partial sequence | uncultured bacterium | 2654 | 2654 | 99 | 0.0 | 98.04 | 1525 | JX898127.1 |
| Alcaligenaceae bacterium BZ45 16S ribosomal RNA gene, partial sequence | Alcaligenaceae bacterium BZ45 | 2654 | 2654 | 99 | 0.0 | 98.04 | 1523 | GQ246952.1 |
| *Pusillimonas ginsengisoli* strain DCY25 16S ribosomal RNA, partial sequence | *Pusillimonas ginsengisoli* | 2647 | 2647 | 95 | 0.0 | 98.99 | 1479 | NR_116103.1 |
| *Pollutimonas nitritireducens* strain JR1/69-2-13 16S ribosomal RNA gene, partial sequence | *Pollutimonas nitritireducens* | 2634 | 2634 | 99 | 0.0 | 97.78 | 1524 | MG205613.2 |
| *Pollutimonas nitritireducens* strain JR1/69-2-13 16S ribosomal RNA, partial sequence | *Pollutimonas nitritireducens* | 2634 | 2634 | 99 | 0.0 | 97.78 | 1524 | NR_184620.1 |
| *Pusillimonas* sp. M17 chromosome, complete genome | *Pusillimonas* sp. M17 | 2634 | 5268 | 99 | 0.0 | 97.60 | 3851523 | CP107548.1 |
| Query: *Dietzia* ANT_WB102 | | | | | | | |  |
| *Dietzia kunjamensis* strain 313 chromosome, complete genome | *Dietzia kunjamensis* | 2782 | 8340 | 100 | 0.0 | 99.61 | 3698013 | CP099712.1 |
| Uncultured bacterium clone YF-24 16S ribosomal RNA gene, partial sequence | uncultured bacterium | 2752 | 2752 | 99 | 0.0 | 99.54 | 1510 | KT905726.1 |
| *Dietzia* sp. BZ84 16S ribosomal RNA gene, partial sequence | *Dietzia* sp. BZ84 | 2750 | 2750 | 99 | 0.0 | 99.54 | 1509 | HQ588860.1 |
| Dietzia kunjamensis subsp. schimae strain YIM 65001 16S ribosomal RNA, partial sequence | *Dietzia kunjamensis* subsp. schimae | 2748 | 2748 | 99 | 0.0 | 99.54 | 1515 | NR_044482.1 |
| Uncultured bacterium clone YF-2 16S ribosomal RNA gene, partial sequence | uncultured bacterium | 2732 | 2732 | 99 | 0.0 | 99.27 | 1512 | KT905711.1 |
| *Dietzia* sp. B32 chromosome | *Dietzia* sp. B32 | 2730 | 8191 | 98 | 0.0 | 99.60 | 3725706 | CP093845.1 |
| *Dietzia* sp. p9(2011) 16S ribosomal RNA gene, partial sequence | *Dietzia* sp. p9(2011) | 2728 | 2728 | 98 | 0.0 | 99.53 | 1497 | HQ652544.1 |
| *Dietzia lutea* strain YIM 80766 chromosome, complete genome | *Dietzia lutea* | 2717 | 10859 | 100 | 0.0 | 98.82 | 3774363 | CP015449.1 |
| *Dietzia* sp. CNJ898 PL04 16S ribosomal RNA gene, partial sequence | *Dietzia* sp. CNJ898 PL04 | 2710 | 2710 | 97 | 0.0 | 99.66 | 1482 | DQ448696.1 |
| *Dietzia* sp. strain VITAPRRKCU-1 16S ribosomal RNA gene, partial sequence | *Dietzia* sp. | 2708 | 2708 | 97 | 0.0 | 99.60 | 1483 | MH118518.1 |
| *Dietzia maris* strain AURCCBT01 16S ribosomal RNA gene, partial sequence | *Dietzia maris* | 2706 | 2706 | 97 | 0.0 | 99.66 | 1483 | MN134494.1 |
| *Dietzia maris* strain 41 16S ribosomal RNA gene, partial sequence | *Dietzia maris* | 2704 | 2704 | 97 | 0.0 | 99.60 | 1483 | KF923451.1 |
| *Dietzia maris* strain AI6 16S ribosomal RNA gene, partial sequence | *Dietzia maris* | 2699 | 2699 | 97 | 0.0 | 99.53 | 1482 | MG547917.1 |
| *Dietzia* sp. A14101 16S ribosomal RNA gene, partial sequence | *Dietzia* sp. A14101 | 2699 | 2699 | 96 | 0.0 | 99.66 | 1477 | AY831396.1 |
| *Dietzia* sp. f5(2011) 16S ribosomal RNA gene, partial sequence | *Dietzia* sp. f5(2011) | 2697 | 2697 | 96 | 0.0 | 99.59 | 1477 | HQ652542.1 |
| *Dietzia psychralcaliphila* strain ILA-1 chromosome, complete genome | *Dietzia psychralcaliphila* | 2693 | 8080 | 100 | 0.0 | 98.56 | 3878247 | CP015453.1 |

# Table S3. Summarize of genes of ANT_WB101 and ANT_WB102 important in bioremediation perspectives.

| Product/Substrate | Enzyme | Gene | EC number | NCBI accession | | KEGG KO |
| --- | --- | --- | --- | --- | --- | --- |
| Genes involved in complex organic compounds degradation | | | | | | |
|  |  |  |  | ANT_WB101 | ANT_WB102 |  |
| Starch | Alpha-amylase | *treS* | 5.4.99.16 3.2.1.1 | WP_149395985.1 | WP_149290605.1 | K05343 |
|  | Isoamylase | *treX* | 3.2.1.68 | WP_149395986.1 | WP_255584368.1 | K01214 |
|  | 4-alpha-glucanotransferase | *malQ* | 2.4.1.25 | WP_149395987.1 | WP_255584186.1 | K00705 |
| Cellulose | Endoglucanase | *bcsZ* | 3.2.1.4 | WP_255474937.1 | - | K20542 |
| Lipids | Triacylglycerol lipase | *lip* | 3.1.1.3 | - | WP_149290821.1,  WP_149291238.1,  WP_149291385.1,  WP_149291386.1, WP_149292460.1 | K01046 |
|  | Alpha/beta hydrolase | *-* | - | WP_149398121.1 | WP_149291991.1,  WP_149291356.1,  WP_149291212.1 | |
|  | Alpha/beta hydrolase | *-* | - | WP_149397978.1 | |  |
|  | Alpha/beta hydrolase | *-* | - | WP_149397977.1 | |  |
| Proteins | Endopeptidase Clp | *clpP* | 3.4.21.92 | WP_149396930.1 | WP_149293042.1, WP_255584313.1 | K01358 |
|  | Endopeptidase La | *lon* | 3.4.21.53 | WP_149396331.1 | - | K01338 |
|  | Peptidase Do | *degP* | 3.4.21.107 | WP_149399767.1 | - | K04771 |
|  | Serine protease DegS | *degS* | 3.4.21.- | WP_149399327.1 | - | K04691 |
|  | Serine-type D-Ala-D-Ala carboxypeptidase | *dacB* | 3.4.21.- | WP_149398711.1 | WP_255583281.1 | K07259 |
|  | Serine-type D-Ala-D-Ala endopeptidase | *pbpG* | 3.4.21.- | WP_149395808.1 | - | K07262 |
| Dissimilatory reduction of nitrate | | | | | | |
| NO_3_^-^ | Nitrate reductase / nitrite oxidoreductase, alpha subunit | narG | 1.7.5.1 1.7.99.- | WP_149396132.1, WP_149397539.1 | WP_149290893.1 | K00370 |
|  | Nitrate reductase / nitrite oxidoreductase, beta subunit | *narH* | 1.7.5.1 1.7.99.- | WP_137238322.1, WP_149397538.1 | WP_149290894.1 | K00371 |
|  | Nitrate reductase gamma subunit | *narI* | 1.7.5.1 1.7.99.- | WP_149396134.1, WP_149397536.1 | WP_149290896.1 | K00374 |
| NO_2_^-^ | Nitrite reductase (NADH) large subunit | *nirB* | 1.7.1.15 | - |  | K00362 |
|  | Nitrite reductase (NADH) small subunit | *nirD* | 1.7.1.15 | - |  | K00363 |
|  | Nitrite reductase (NO-forming) | *nirK* | 1.7.2.1 | WP_149399201.1 | WP_255583382.1 | K00368 |
| NO | Nitric oxide reductase subunit B | *norB* | 1.7.2.5 | WP_149396017.1, WP_149396870.1, WP_149397000.1, WP_223146238.1 | - | K04561 |
| N_2_O | Nitrous-oxide reductase | *nosZ* | 1.7.2.4 | WP_149399658.1 | - | K00376 |
| Genes involved in xenobiotics degradation | | | | | | |
| Halogen compounds | 2-haloacid dehalogenase | E3.8.1.2 | 3.8.1.2 | WP_149396064.1, WP_149397276.1 | - | K01560 |
|  | Haloalkane dehalogenase | *dhaA* | 3.8.1.5 | WP_149396890.1 | - | K01563 |
| Hydrogen peroxide | Catalase | *katE* | 1.11.1.6 | WP_149398152.1, WP_188074590.1 | WP_149290721.1 | K03781 |
| Organic compounds | Chloride peroxidase | *-* | 1.11.1.10 | - | WP_255583668.1 | K00433 |
| Reactive Blue 5 | Dye decolorizing peroxidase | *-* | K15733 | - | WP_149291414.1 | K15733 |
| Propane/phenol | Propane 2-monooxygenase | *prmA* | 1.14.13.227 | - | WP_149291033.1 | K18223 |
|  | Propane 2-monooxygenase | *prmC* | - | - | WP_149292607.1 | K18224 |
|  | Propane monooxygenase reductase component | *prmB* | 1.18.1.- | WP_149399445.1 | WP_149291034.1 | K18225 |
| Alkane degradation | Cytochrome P450 (**CYP153**) | *p450* | - | WP_223146200.1, WP_149399654.1 | WP_255584093.1,  **WP_149291016.1**,  WP_149291230.1,  **WP_149291686.1**,  **WP_149291793.1**,  WP_149292702.1,  **WP_149292015.1**,  **WP_149292018.1**,  **WP_149293469.1** | |
|  | Alkane monooxygenase | *alkB/ladA* | - | WP_149396949.1 | FQ137_RS15475 (KAA0918250.1) | |
|  | Rubredoxin | *alkG* | - | WP_149398089.1 | WP_255584096.1 |  |
| Phenols | Polyphenol oxidase | *pgeF* | - | WP_149399682.1 | WP_149293172.1 |  |

# Table S4. The results of analysis of resistome of ANT_WB101 and ANT_WB102 using RGI Database.

| Accession | Identity [%] | Cut_Off | Best_Hit_ARO | Drug |
| --- | --- | --- | --- | --- |
| ANT_WB101 | | | | |
| WP_149396566.1, WP_149397660.1, WP_255474979.1 | 28.47 - 36.62 | Loose | novA | Aminocoumarin |
| WP_149397830.1 | 45.89 | Loose | mdtA | Aminocoumarin |
| WP_149396312.1 | 31.33 | Loose | apmA | Aminoglycoside |
| WP_149395455.1, WP_149395644.1, WP_149395820.1, WP_149395827.1, WP_149395944.1, WP_149396081.1, WP_149396148.1, WP_149396189.1, WP_149396201.1, WP_149396209.1, WP_149396340.1, WP_149396431.1, WP_149397231.1, WP_149397264.1, WP_149397265.1, WP_149397279.1, WP_149397404.1, WP_149397593.1, WP_149397894.1, WP_149397897.1, WP_149398038.1, WP_149398090.1, WP_149398195.1, WP_149398480.1, WP_149398629.1, WP_149398921.1, WP_149399094.1, WP_149399194.1, WP_149399274.1, WP_149399292.1, WP_149399536.1, WP_149399555.1, WP_149399738.1, WP_255474897.1, WP_255475049.1 | 23.73 - 34.09 | Loose | RanA | Aminoglycoside |
| WP_149397278.1, WP_149399294.1 | 28 - 28.11 | Loose | RanB | Aminoglycoside |
| WP_149398966.1, WP_188074523.1 | 27.6 - 31.03 | Loose | kdpD | Aminoglycoside |
| WP_149397329.1, WP_149398967.1 | 34.55 - 61.36 | Loose | kdpE | Aminoglycoside |
| WP_149396054.1 | 31.36 | Loose | CrcB | Aminoglycoside |
| WP_149395255.1 | 25.45 | Loose | smeS | Aminoglycoside |
| WP_149397205.1, WP_149397494.1, WP_188074641.1, WP_255475104.1 | 23.71 - 31.02 | Loose | cpxA | Aminoglycoside |
| WP_149395685.1, WP_149395913.1, WP_149397851.1, WP_149398335.1 | 25.84 - 31.54 | Loose | baeS | Aminoglycoside |
| WP_149397763.1, WP_149398190.1, WP_149398543.1 | 31.86 - 40 | Loose | baeR | Aminoglycoside |
| WP_149395776.1 | 26.18 | Loose | farA | Antibacterial |
| WP_149395615.1 | 49.22 | Loose | farB | Antibacterial |
| WP_149399374.1 | 32.73 | Loose | bcr-1 | Bicyclomycin-like |
| WP_255474889.1 | 35.62 | Loose | CAR-1 | Carbapenem |
| WP_149395626.1, WP_149398063.1 | 28.18 - 29.85 | Loose | DHT2-1 | Carbapenem |
| WP_149397615.1 | 26.01 | Loose | MSI-1 | Carbapenem |
| WP_149396458.1 | 27.16 | Loose | ACT-83 | Carbapenem; |
| WP_149395286.1, WP_149395371.1, WP_149396217.1, WP_149396246.1, WP_149396255.1, WP_149396510.1, WP_149396528.1, WP_149396631.1, WP_149396660.1, WP_149397072.1, WP_149397125.1, WP_149397352.1, WP_149397388.1, WP_149397509.1, WP_149397586.1, WP_149398553.1, WP_149398568.1, WP_149398616.1, WP_255474935.1, WP_255474962.1 | 24.57 - 37.97 | Loose | NmcR | Carbapenem; |
| WP_149395316.1 | 24.75 | Loose | PJM-1 | Carbapenem; |
| WP_149398769.1 | 39.14 | Loose | Neisseria | Cephalosporin; |
| WP_149396639.1, WP_149398250.1 | 24.22 - 33.9 | Loose | Helicobacter | Cephalosporin; |
| WP_149395272.1 | 46.91 | Loose | dfrA3 | Diaminopyrimidine |
| WP_149395778.1, WP_149396520.1, WP_149397274.1, WP_149398016.1, WP_149398257.1, WP_149399106.1, WP_149399776.1 | 23.17 - 43.48 | Loose | Escherichia | Disinfecting |
| WP_149396450.1 | 27.24 | Loose | TriA | Disinfecting |
| WP_149397843.1 | 32.96 | Loose | OpmH | Disinfecting |
| WP_149396790.1 | 36 | Loose | qacJ | Disinfecting |
| WP_149395983.1 | 24.91 | Loose | Planobispora | Elfamycin |
| WP_149398176.1 | 81.77 | Loose | Escherichia | Elfamycin |
| WP_149398607.1 | 33.33 | Loose | Escherichia | Fluoroquinolone |
| WP_149396991.1, WP_188074537.1 | 26.57 - 33.59 | Loose | mgrA | Fluoroquinolone |
| WP_149398140.1 | 59.63 | Loose | Morganella | Fluoroquinolone |
| WP_255474882.1, WP_149396704.1, WP_255474884.1 | 35.49 - 60.74 | Loose | Clostridioides | Fluoroquinolone |
| WP_149396563.1 | 20.36 | Loose | Staphylococcus | Fluoroquinolone |
| WP_149396427.1 | 25.28 | Loose | sdrM | Fluoroquinolone |
| WP_149397286.1 | 25.45 | Loose | arlS | Fluoroquinolone |
| WP_255474994.1 | 27.09 | Loose | mdeA | Fluoroquinolone |
| WP_149398974.1 | 27.84 | Loose | lfrA | Fluoroquinolone |
| WP_149398255.1 | 32.63 | Loose | hp1181 | Fluoroquinolone |
| WP_149395616.1 | 46.76 | Loose | emrA | Fluoroquinolone |
| WP_149397217.1, WP_255474780.1 | 23.27 - 24.71 | Loose | blt | Fluoroquinolone |
| WP_149396191.1, WP_255474968.1, WP_255475068.1 | 27.56 - 33.77 | Loose | emrR | Fluoroquinolone |
| WP_149396113.1, WP_149396634.1, WP_149397287.1, WP_149398247.1, WP_149398475.1, WP_149398972.1 | 30.88 - 40.74 | Loose | arlR | Fluoroquinolone |
| WP_255474795.1 | 28.82 | Loose | hmrM | Fluoroquinolone |
| WP_149396481.1 | 33.48 | Loose | abeM | Fluoroquinolone |
| WP_149397336.1 | 18.93 | Loose | AcrS | Fluoroquinolone |
| WP_255474949.1 | 31.03 | Loose | OpmD | Fluoroquinolone |
| WP_149398161.1 | 35.29 | Loose | marA | Fluoroquinolone |
| WP_149396416.1 | 46.68 | Loose | acrB | Fluoroquinolone |
| WP_149397578.1 | 53.72 | Loose | adeG | Fluoroquinolone |
| WP_149398806.1 | 54.76 | Loose | YajC | Fluoroquinolone |
| WP_149397576.1 | 57.42 | Loose | adeH | Fluoroquinolone |
| WP_149395855.1, WP_149396317.1, WP_149396811.1, WP_149396839.1, WP_149397233.1, WP_149397237.1, WP_149397303.1, WP_149397454.1, WP_149397562.1, WP_149398018.1, WP_149398119.1, WP_149399475.1, WP_149399920.1, WP_188074606.1 | 23.62 - 39.08 | Loose | MexS | Fluoroquinolone |
| WP_149395366.1, WP_149395431.1, WP_149395483.1, WP_149395620.1, WP_149395839.1, WP_149396766.1, WP_149397075.1, WP_149397096.1, WP_149397212.1, WP_149397285.1, WP_149397295.1, WP_149397340.1, WP_149397466.1, WP_149397487.1, WP_149397579.1, WP_149397922.1, WP_149397954.1, WP_149398081.1, WP_149398127.1, WP_149398944.1, WP_149398995.1, WP_149399053.1, WP_149399091.1, WP_149399115.1, WP_149399372.1, WP_149399505.1, WP_223146236.1, WP_255475080.1 | 24.35 - 58.21 | Loose | adeL | Fluoroquinolone |
| WP_149395315.1, WP_149398279.1, WP_255475017.1 | 27.27 - 31.15 | Loose | MexT | Fluoroquinolone |
| WP_149396553.1, WP_149397159.1 | 29.89 - 31.46 | Loose | ramA | Fluoroquinolone |
| WP_149395618.1, WP_149397192.1, WP_188074671.1, WP_255474926.1, WP_149395904.1 | 32.31 - 46.45 | Loose | Escherichia | Fluoroquinolone |
| WP_149397157.1, WP_149398192.1, WP_149399413.1 | 27.93 - 59.83 | Loose | Staphylococcus | Fusidane |
| WP_149397227.1 | 27.43 | Loose | vanG | Glycopeptide |
| WP_149395657.1, WP_149396507.1, WP_149397707.1, WP_149397737.1, WP_149397850.1, WP_149398431.1, WP_149398960.1, WP_188074596.1 | 25.24 - 41.38 | Loose | vanR | Glycopeptide |
| WP_149399565.1, WP_188074593.1 | 27.22 - 28.68 | Loose | vanT | Glycopeptide |
| WP_149398762.1 | 35.15 | Loose | D-Ala-D-Ala | Glycopeptide |
| WP_149396212.1, WP_149396239.1, WP_149396249.1, WP_149396264.1, WP_149396392.1, WP_149396543.1, WP_149396886.1, WP_149397503.1, WP_149398031.1, WP_149399479.1, WP_255474832.1, WP_255475045.1 | 27.14 - 36.32 | Loose | vanH | Glycopeptide |
| WP_149395818.1, WP_149397542.1, WP_149398188.1 | 27.31 - 28.28 | Loose | vanS | Glycopeptide |
| WP_149398258.1 | 35.47 | Loose | vanX | Glycopeptide |
| WP_149396974.1 | 31.79 | Loose | cfrC | Lincosamide |
| WP_149397716.1, WP_149398725.1 | 29 - 31.34 | Loose | lmrC | Lincosamide |
| WP_149398818.1 | 35.09 | Loose | macA | Macrolide |
| WP_149395472.1, WP_149396069.1, WP_149396106.1, WP_149396151.1, WP_149396449.1, WP_149396665.1, WP_149396768.1, WP_149396799.1, WP_149396914.1, WP_149396915.1, WP_149397114.1, WP_149397333.1, WP_149397346.1, WP_149397431.1, WP_149397455.1, WP_149397524.1, WP_149397791.1, WP_149397807.1, WP_149398254.1, WP_149398559.1, WP_149398609.1, WP_149398622.1, WP_149398655.1, WP_149398742.1, WP_149398747.1, WP_149398796.1, WP_149398880.1, WP_149398883.1, WP_149398911.1, WP_149399172.1, WP_149399195.1, WP_149399398.1, WP_149399900.1, WP_188074622.1, WP_223146208.1, WP_223146212.1, WP_223146256.1, WP_255474859.1, WP_255474942.1, WP_255475014.1, WP_255475063.1 | 19.75 - 41.33 | Loose | macB | Macrolide |
| WP_149395301.1, WP_149395333.1, WP_149395836.1, WP_149395898.1, WP_149396371.1, WP_149396561.1, WP_149397154.1, WP_149397173.1, WP_149397753.1 | 24.68 - 33.33 | Loose | oleC | Macrolide |
| WP_149398809.1 | 27.82 | Loose | Erm(30) | Macrolide |
| WP_188074489.1 | 33.33 | Loose | ErmO-srmA | Macrolide |
| WP_149397842.1 | 41.27 | Loose | ErmB | Macrolide |
| WP_149398393.1 | 33.62 | Loose | mphO | Macrolide |
| WP_149396366.1 | 29.04 | Loose | mreA | Macrolide |
| WP_149395303.1, WP_149397534.1, WP_149397833.1, WP_149398499.1 | 21.95 - 32.76 | Loose | evgA | Macrolide |
| WP_149395658.1, WP_149395783.1, WP_149397918.1, WP_149398107.1, WP_149398248.1 | 25.51 - 34.17 | Loose | evgS | Macrolide |
| WP_149398093.1 | 33.45 | Loose | RlmA(II) | Macrolide |
| WP_149397951.1 | 22.78 | Loose | adeN | Macrolide |
| WP_149395498.1 | 26.53 | Loose | MexR | Macrolide |
| WP_149399098.1 | 30.53 | Loose | OprZ | Macrolide |
| WP_149398816.1 | 33.55 | Loose | smeF | Macrolide |
| WP_149398222.1 | 33.93 | Loose | nalD | Macrolide |
| WP_188074670.1 | 34.69 | Loose | MexJ | Macrolide |
| WP_149397612.1 | 35.5 | Loose | MexV | Macrolide |
| WP_149397611.1 | 41.03 | Loose | MexW | Macrolide |
| WP_149396415.1 | 41.82 | Loose | AxyX | Macrolide |
| WP_188074634.1 | 50.21 | Loose | OpmB | Macrolide |
| WP_149399100.1 | 53.85 | Loose | MexK | Macrolide |
| WP_149397828.1 | 57.38 | Loose | MuxC | Macrolide |
| WP_149397829.1 | 65.65 | Loose | MuxB | Macrolide |
| WP_149396473.1, WP_149396713.1, WP_149397898.1, WP_149398059.1, WP_149399203.1 | 22.89 - 30.51 | Loose | CRP | Macrolide |
| WP_149395779.1, WP_149397986.1 | 27.97 - 33.33 | Loose | MexL | Macrolide |
| WP_149398433.1, WP_149398500.1, WP_149398504.1 | 28.15 - 31.25 | Loose | ParS | Macrolide |
| WP_149396555.1, WP_188074594.1 | 29.25 - 29.7 | Loose | mtrA | Macrolide |
| WP_149397887.1, WP_149399858.1 | 34.17 - 38.16 | Loose | Pseudomonas | Macrolide |
| WP_149395777.1, WP_255474905.1 | 45.42 - 45.97 | Loose | OprM | Macrolide |
| WP_149399894.1 | 30.21 | Loose | Burkholderia | Monobactam; |
| WP_149395635.1, WP_149395975.1 | 33.06 - 53.97 | Loose | golS | Monobactam; |
| WP_149395511.1 | 21.88 | Loose | Bifidobacterium | Mupirocin-like |
| WP_149396367.1, WP_149396689.1 | 22.01 - 27.28 | Loose | Staphylococcus | Mupirocin-like |
| WP_149396314.1, WP_149399516.1 | 26.55 - 39.41 | Loose | msbA | Nitroimidazole |
| WP_149395617.1 | 32.22 | Loose | mdtP | Nucleoside |
| WP_149395486.1, WP_149395623.1, WP_149395824.1, WP_149396280.1, WP_149397382.1, WP_149398264.1, WP_188074666.1 | 17.86 - 30.77 | Loose | leuO | Nucleoside |
| WP_149399857.1 | 28.4 | Loose | SatA | Nucleoside |
| WP_149399488.1 | 40.91 | Loose | sta | Nucleoside |
| WP_149395865.1, WP_149396353.1, WP_149397247.1 | 28.84 - 32.84 | Loose | optrA | Oxazolidinone |
| WP_255475069.1 | 23.43 | Loose | mecC | Penam |
| WP_188074659.1 | 27.6 | Loose | LptD | Peptide |
| WP_149395456.1, WP_149395775.1, WP_149395835.1, WP_149396836.1, WP_149397663.1, WP_149397806.1, WP_149397861.1, WP_149397972.1, WP_149398037.1, WP_149398045.1, WP_149398623.1, WP_149398660.1, WP_149399272.1, WP_149399827.1, WP_223146300.1 | 26.5 - 35.81 | Loose | bcrA | Peptide |
| WP_149396725.1 | 37.44 | Loose | Staphylococcus | Peptide |
| WP_255474966.1, WP_149395391.1, WP_149397337.1, WP_149397573.1, WP_149397708.1, WP_149398120.1 | 23.3 - 30.1 | Loose | Enterococcus | Peptide |
| WP_149396092.1 | 36.07 | Loose | Brucella | Peptide |
| WP_149395780.1, WP_149396753.1, WP_149397760.1, WP_255475089.1 | 35.54 - 43.44 | Loose | OmpA | Peptide |
| WP_149396829.1, WP_149399524.1 | 22.56 - 26.29 | Loose | LpsB | Peptide |
| WP_149397540.1 | 23.36 | Loose | rosA | Peptide |
| WP_149395786.1, WP_149395936.1 | 27.03 - 29.65 | Loose | tsnR | Peptide |
| WP_149397889.1 | 27.51 | Loose | arnA | Peptide |
| WP_149395252.1 | 29.73 | Loose | ArnT | Peptide |
| WP_149396114.1, WP_149396635.1, WP_149397328.1, WP_149397736.1, WP_149398155.1, WP_149398476.1, WP_149398924.1, WP_149398961.1, WP_149398971.1, WP_223146182.1, WP_255474867.1 | 23.61 - 40.58 | Loose | basS | Peptide |
| WP_149395253.1, WP_149398560.1, WP_255474969.1 | 27.17 - 31.21 | Loose | PmrF | Peptide |
| WP_149395256.1, WP_149398503.1, WP_149398925.1 | 38.36 - 39.19 | Loose | cprR | Peptide |
| WP_149395304.1, WP_149398215.1, WP_149398216.1, WP_188074640.1, WP_255474873.1, WP_255474941.1 | 21.94 - 30.34 | Loose | almE | Peptide |
| WP_149395419.1 | 37.28 | Loose | bacA | Peptide |
| WP_149395726.1, WP_149397294.1, WP_223146228.1 | 24.21 - 36.06 | Loose | cmlv | Phenicol |
| WP_149397335.1 | 29.62 | Loose | mexM | Phenicol |
| WP_149399304.1 | 51.07 | Loose | Staphylococcus | Phosphonic |
| WP_149395766.1, WP_149395912.1, WP_149397206.1, WP_149398432.1 | 28.79 - 33.67 | Loose | Escherichia | Phosphonic |
| WP_149398686.1 | 32.52 | Loose | FosC2 | Phosphonic |
| WP_149398096.1 | 23.75 | Loose | mdtG | Phosphonic |
| WP_149396967.1, WP_149397500.1, WP_255474961.1 | 28.61 - 39.51 | Loose | Acinetobacter | Phosphonic |
| WP_149398556.1, WP_149398772.1 | 35.04 - 39.96 | Loose | TaeA | Pleuromutilin |
| WP_149395938.1 | 34.19 | Loose | rphB | Rifamycin |
| WP_149396422.1, WP_149398531.1 | 24.44 - 29.21 | Loose | Nocardia | Rifamycin |
| WP_149398418.1, WP_149399125.1 | 25.91 - 29.31 | Loose | Streptomyces | Rifamycin |
| WP_149398183.1 | 57 | Loose | Mycobacterium | Rifamycin |
| WP_149396268.1 | 30.09 | Loose | vatE | Streptogramin |
| WP_149398636.1 | 41.76 | Loose | sul4 | Sulfonamide |
| WP_149398261.1 | 29.87 | Loose | tetA(46) | Tetracycline |
| WP_149397523.1 | 32.88 | Loose | tetB(46) | Tetracycline |
| WP_149395633.1, WP_149395819.1, WP_149397903.1, WP_149397904.1, WP_149398986.1, WP_149399717.1 | 37.62 - 44.21 | Loose | TxR | Tetracycline |
| WP_149398262.1 | 25.45 | Loose | tet(V) | Tetracycline |
| WP_149395497.1, WP_149397913.1 | 25.18 - 32.94 | Loose | otr(B) | Tetracycline |
| WP_149395862.1, WP_149396769.1, WP_149396876.1, WP_149397153.1, WP_149397424.1, WP_149397432.1, WP_149397433.1, WP_149397560.1, WP_149397561.1, WP_149398008.1, WP_149398009.1, WP_149398148.1, WP_149398149.1, WP_149398380.1, WP_149398386.1, WP_149398387.1, WP_149398600.1, WP_149398610.1, WP_149398647.1, WP_149398989.1, WP_149399300.1, WP_149399660.1, WP_149399825.1, WP_149399835.1, WP_149399837.1 | 26.69 - 40.57 | Loose | tetA(58) | Tetracycline |
| WP_149395257.1, WP_149398469.1 | 30.99 - 34.71 | Loose | tcr3 | Tetracycline |
| WP_149399648.1 | 29.33 | Loose | tet(O/32/O) | Tetracycline |
| WP_149399642.1, WP_149399765.1 | 41.5 - 47.18 | Loose | Streptomyces | Tetracycline |
| WP_149398416.1 | 42.45 | Strict | qacG | Disinfecting |
| WP_149395905.1, WP_149397577.1 | 42.75 - 79.94 | Strict | adeF | Fluoroquinolone |
| ANT_WB102 | | | | |
| WP_149291653.1, WP_149292392.1, WP_149292599.1 | 28.32 - 36.82 | Loose | novA | Aminocoumarin |
| WP_149292856.1 | 29.7 | Loose | aacA43 | Aminoglycoside |
| WP_255583275.1 | 25.48 | Loose | APH(2'')-IVa | Aminoglycoside |
| WP_149291767.1, WP_149293449.1, WP_149293450.1, WP_188064974.1 | 25.32 - 28.78 | Loose | RanB | Aminoglycoside |
| WP_149291087.1, WP_149291194.1, WP_149291387.1, WP_149291444.1, WP_149291766.1, WP_255584114.1 | 27.39 - 34.44 | Loose | RanA | Aminoglycoside |
| WP_255583581.1 | 31.63 | Loose | cpxA | Aminoglycoside |
| WP_188065017.1 | 43.5 | Loose | smeR | Aminoglycoside |
| WP_149290688.1, WP_149291367.1, WP_149293426.1 | 26.89 - 36.33 | Loose | baeS | Aminoglycoside |
| WP_149292950.1, WP_255583798.1 | 28.95 - 30.1 | Loose | smeS | Aminoglycoside |
| WP_255583897.1 | 33.77 | Loose | bcr-1 | Bicyclomycin-like |
| WP_188065045.1 | 25.69 | Loose | EAM-1 | Carbapenem |
| WP_149291147.1, WP_149291221.1 | 34.48 | Loose | ELM-1 | Carbapenem |
| WP_255583861.1 | 31.51 | Loose | GRD33-1 | Carbapenem |
| WP_149292789.1 | 29.27 | Loose | POM-1 | Carbapenem |
| WP_149293253.1 | 26.16 | Loose | SPG-1 | Carbapenem |
| WP_149292992.1 | 22.64 | Loose | LHK-1 | Carbapenem; |
| WP_149293095.1, WP_188065030.1 | 30.37 - 34.17 | Loose | LHK-3 | Carbapenem; |
| WP_149292473.1 | 36.84 | Loose | NmcR | Carbapenem; |
| WP_149291928.1 | 28.91 | Loose | PAC-1 | Cephalosporin |
| WP_149291113.1 | 26.21 | Loose | Helicobacter | Cephalosporin; |
| WP_188065040.1 | 28.81 | Loose | Neisseria | Cephalosporin; |
| WP_149291981.1 | 27.84 | Loose | CMY-114 | Cephamycin |
| WP_149291872.1 | 42.75 | Loose | dfrA3 | Diaminopyrimidine |
| WP_149291691.1, WP_149290663.1, WP_149291707.1, WP_149291790.1, WP_149292409.1 | 23.83 - 34.36 | Loose | Escherichia | Disinfecting |
| WP_149291818.1 | 73.23 | Loose | Escherichia | Elfamycin |
| WP_149291715.1, WP_188064747.1 | 28.57 - 30.07 | Loose | Planobispora | Elfamycin |
| WP_149292191.1, WP_255583295.1 | 33.05 - 41.01 | Loose | facT | Elfamycin |
| WP_149292456.1 | 34.78 | Loose | mgrA | Fluoroquinolone |
| WP_149292509.1 | 38.71 | Loose | Pseudomonas | Fluoroquinolone |
| WP_149290986.1 | 46.8 | Loose | Clostridioides | Fluoroquinolone |
| WP_149292143.1 | 25.38 | Loose | bmr | Fluoroquinolone |
| WP_149290984.1 | 26.53 | Loose | norA | Fluoroquinolone |
| WP_149291079.1 | 27.01 | Loose | mdeA | Fluoroquinolone |
| WP_149291606.1 | 29.76 | Loose | lfrA | Fluoroquinolone |
| WP_149291366.1, WP_188064977.1 | 39.64 - 40.36 | Loose | arlR | Fluoroquinolone |
| WP_255583956.1 | 25.49 | Loose | PmpM | Fluoroquinolone |
| WP_149291380.1 | 26.36 | Loose | QnrB2 | Fluoroquinolone |
| WP_149290808.1, WP_149290812.1, WP_149291038.1, WP_149291309.1, WP_149291554.1, WP_149292012.1, WP_149292385.1, WP_149292728.1, WP_255583858.1 | 26.43 - 37.68 | Loose | MexS | Fluoroquinolone |
| WP_149291084.1, WP_149291702.1, WP_149291815.1 | 28.24 - 38.98 | Loose | Escherichia | Fluoroquinolone |
| WP_149290574.1, WP_149291190.1 | 29.7 - 42 | Loose | AcrS | Fluoroquinolone |
| WP_149291936.1 | 26.16 | Loose | Staphylococcus | Fusidane |
| WP_149291041.1 | 24.89 | Loose | vanM | Glycopeptide |
| WP_149291892.1 | 30.84 | Loose | vanO | Glycopeptide |
| WP_149292852.1 | 31.52 | Loose | vanT | Glycopeptide |
| WP_149292219.1 | 37.5 | Loose | vanB | Glycopeptide |
| WP_149291282.1, WP_149291567.1, WP_149293399.1, WP_149293486.1 | 35.96 - 40.61 | Loose | vanR | Glycopeptide |
| WP_149290615.1, WP_149292203.1, WP_255583495.1 | 35.09 - 35.54 | Loose | vanH | Glycopeptide |
| WP_149291566.1 | 34.05 | Loose | vanS | Glycopeptide |
| WP_149291424.1 | 27.16 | Loose | vanW | Glycopeptide |
| WP_149291669.1 | 23.3 | Loose | Mycolicibacterium | Isoniazid-like |
| WP_149291192.1 | 45.34 | Loose | Mycobacterium | Isoniazid-like |
| WP_149291994.1 | 35.67 | Loose | lmrB | Lincosamide |
| WP_149292259.1 | 37.13 | Loose | clbC | Lincosamide |
| WP_149291557.1 | 41.41 | Loose | lmrC | Lincosamide |
| WP_149291157.1, WP_149291259.1, WP_149291401.1, WP_149291696.1, WP_149291733.1, WP_149291749.1, WP_149292092.1, WP_149292107.1, WP_149292455.1, WP_149292534.1, WP_149292631.1, WP_149292989.1, WP_149293394.1, WP_188065049.1, WP_255583642.1 | 27.08 - 42.42 | Loose | macB | Macrolide |
| WP_149290814.1, WP_149291599.1, WP_149291600.1, WP_149292177.1, WP_149292625.1, WP_255583540.1 | 29.82 - 36.44 | Loose | oleC | Macrolide |
| WP_149292719.1 | 29.09 | Loose | ErmR | Macrolide |
| WP_255583957.1 | 31.86 | Loose | mreA | Macrolide |
| WP_149291154.1, WP_149291960.1, WP_149292868.1 | 23.76 - 26.82 | Loose | evgA | Macrolide |
| WP_149293403.1 | 35.71 | Loose | carA | Macrolide |
| WP_149290628.1, WP_149293258.1 | 32.66 - 33.45 | Loose | tlrC | Macrolide |
| WP_149292397.1, WP_149293254.1 | 32.69 - 33.58 | Loose | oleB | Macrolide |
| WP_149293011.1 | 33.04 | Loose | msrH | Macrolide |
| WP_255583919.1 | 35.38 | Loose | myrA | Macrolide |
| WP_149292140.1 | 21.43 | Loose | nalD | Macrolide |
| WP_188064849.1 | 28.92 | Loose | gadX | Macrolide |
| WP_149291323.1 | 30 | Loose | Type | Macrolide |
| WP_063972214.1 | 31.94 | Loose | CRP | Macrolide |
| WP_188064795.1 | 37.5 | Loose | cmeR | Macrolide |
| WP_149292403.1 | 39.19 | Loose | nalC | Macrolide |
| WP_149291358.1, WP_149291395.1, WP_149291886.1, WP_149291946.1, WP_255583790.1 | 23.24 - 44.9 | Loose | adeN | Macrolide |
| WP_149292039.1, WP_149292967.1 | 28.93 - 37.7 | Loose | MexL | Macrolide |
| WP_149293019.1, WP_188064757.1 | 32.53 - 34.62 | Loose | MexZ | Macrolide |
| WP_149292717.1 | 32.71 | Loose | abeS | Macrolide |
| WP_255583801.1 | 33.94 | Loose | Klebsiella | Macrolide |
| WP_149290862.1, WP_149292652.1 | 31.03 - 32.31 | Loose | golS | Monobactam; |
| WP_149293045.1, WP_188064737.1 | 22.68 - 27.97 | Loose | Staphylococcus | Mupirocin-like |
| WP_149291919.1, WP_255584391.1 | 47.17 - 53.31 | Loose | Bifidobacterium | Mupirocin-like |
| WP_149293404.1 | 22.18 | Loose | msbA | Nitroimidazole |
| WP_149293157.1 | 37.35 | Loose | sta | Nucleoside |
| WP_149290964.1 | 26.29 | Loose | mecA | Penam |
| WP_149291332.1, WP_149291508.1 | 34.93 - 36.6 | Loose | bcrA | Peptide |
| WP_255584203.1, WP_149290865.1 | 25.12 - 25.16 | Loose | Enterococcus | Peptide |
| WP_255583389.1 | 23.22 | Loose | Bacillus | Peptide |
| WP_149291563.1, WP_149292084.1 | 24.19 - 25.08 | Loose | LpsB | Peptide |
| WP_149292099.1 | 24.71 | Loose | rosB | Peptide |
| WP_149290630.1, WP_149292833.1, WP_255584386.1 | 25.68 - 31.58 | Loose | tsnR | Peptide |
| WP_149291756.1, WP_149292803.1 | 25.78 - 26.81 | Loose | PmrF | Peptide |
| WP_149292365.1, WP_149292712.1 | 31.67 - 35.45 | Loose | arnA | Peptide |
| WP_149290579.1, WP_149290664.1, WP_149292181.1 | 23.4 - 28.64 | Loose | almE | Peptide |
| WP_149292801.1 | 34.18 | Loose | bacA | Peptide |
| WP_149290902.1 | 34.62 | Loose | bcrC | Peptide |
| WP_149291619.1 | 33.33 | Loose | Agrobacterium | Phenicol |
| WP_149291627.1 | 39.02 | Loose | catB11 | Phenicol |
| WP_149291887.1, WP_149292888.1, WP_188065089.1 | 24.28 - 36.66 | Loose | cmlv | Phenicol |
| WP_149290904.1, WP_255583548.1 | 23.19 - 26.78 | Loose | fexA | Phenicol |
| WP_149293336.1 | 61.59 | Loose | Mycobacterium | Phosphonic |
| WP_149291134.1, WP_149291803.1, WP_149292041.1, WP_255583500.1 | 29.74 - 35.25 | Loose | Acinetobacter | Phosphonic |
| WP_149291923.1, WP_149293028.1 | 39.48 - 39.78 | Loose | TaeA | Pleuromutilin |
| WP_149290825.1, WP_149290826.1, WP_149293341.1 | 42.44 - 45.48 | Loose | Mycobacterium | Polyamine |
| WP_149292282.1 | 33.64 | Loose | Mycobacterium | Pyrazine |
| WP_149291982.1, WP_149293251.1 | 32.51 - 58.28 | Loose | HelR | Rifamycin |
| WP_149292453.1 | 77.27 | Loose | RbpA | Rifamycin |
| WP_223146507.1 | 27.03 | Loose | Nocardia | Rifamycin |
| WP_149291961.1 | 39.75 | Loose | rphA | Rifamycin |
| WP_149291776.1 | 82.35 | Loose | Mycobacterium | Rifamycin |
| WP_149291873.1 | 79.77 | Loose | Mycobacterium | Salicylic |
| WP_149291281.1 | 36.51 | Loose | VatI | Streptogramin |
| WP_149292537.1, WP_149292756.1 | 40.15 - 41.11 | Loose | sul4 | Sulfonamide |
| WP_149291032.1 | 28.46 | Loose | TxR | Tetracycline |
| WP_149292723.1 | 27.95 | Loose | tet(V) | Tetracycline |
| WP_149290733.1 | 30.18 | Loose | tetR | Tetracycline |
| WP_149291857.1 | 35.55 | Loose | tetB(58) | Tetracycline |
| WP_149292649.1, WP_255584380.1 | 23.37 - 40.46 | Loose | otr(B) | Tetracycline |
| WP_149290953.1, WP_149291245.1, WP_149292210.1 | 25.79 - 35.56 | Loose | tcr3 | Tetracycline |
| WP_149290936.1, WP_149291150.1 | 25.95 - 27.96 | Loose | tet(41) | Tetracycline |
| WP_149291166.1, WP_149291175.1, WP_149291664.1, WP_149291856.1, WP_149292387.1, WP_149292419.1, WP_149292878.1, WP_149293093.1, WP_149293213.1, WP_149293323.1, WP_255583299.1 | 29.1 - 50.15 | Loose | tetA(58) | Tetracycline |
| WP_149291817.1 | 26.62 | Loose | tet(T) | Tetracycline |
| WP_149292112.1 | 28.48 | Loose | Streptomyces | Tetracycline |
| WP_149292275.1 | 31.11 | Loose | tet(36) | Tetracycline |
| WP_149293071.1 | 34.87 | Loose | tetB(P) | Tetracycline |
| WP_149292265.1 | 25.63 | Loose | Mycobacterium | Thioamide |
| WP_188065025.1 | 59.18 | Strict | Mycobacterium | Salicylic |

# Table S5. The results of analysis of virulence factors ANT_WB101 and ANT_WB102 using VFDB.

| Accessions | Identity [%] | VFG | VF category | VF_name |
| --- | --- | --- | --- | --- |
| ANT_WB101 | | | | |
| WP_149396473.1, WP_149396713.1, WP_149396952.1, WP_149397898.1, WP_149398059.1, WP_149398910.1, WP_149399203.1 | 21.88 - 26.59 | VFG042734 | Adherence | Type IV pili |
| WP_149395485.1, WP_149396181.1, WP_149396961.1, WP_149397584.1, WP_149397589.1, WP_149397590.1, WP_149397947.1, WP_149397975.1, WP_149398030.1, WP_149398136.1 | 24.16 - 33.33 | VFG006717 | Adherence | Lap |
| WP_149395894.1, WP_149398900.1 | 25 - 40.7 | VFG042739 | Adherence | Type IV pili |
| WP_149395983.1, WP_149397157.1, WP_149398192.1, WP_149399413.1, WP_149399642.1, WP_149399648.1, WP_149399765.1 | 25 - 31.22 | VFG046465 | Adherence | EF-Tu |
| WP_149396109.1, WP_149396451.1, WP_149396666.1, WP_149397309.1, WP_149397458.1, WP_149398882.1, WP_149399902.1, WP_255474893.1 | 24.48 - 30.95 | VFG001983 | Adherence | PEB1 |
| WP_149396377.1 | 27.73 | VFG045345 | Adherence | MAM7 |
| WP_149396396.1 | 27.37 | VFG048237 | Adherence | Type I fimbriae |
| WP_149396445.1 | 29.01 | VFG000457 | Adherence | Agf |
| WP_149396488.1 | 28.15 | VFG051131 | Adherence | BmaC |
| WP_149396553.1 | 31.33 | VFG001234 | Adherence | Type IV pili |
| WP_149396591.1 | 24.71 | VFG001230 | Adherence | Type IV pili |
| WP_149396614.1 | 27.74 | VFG038529 | Adherence | Flp type IV pili |
| WP_149396700.1 | 28.06 | VFG002434 | Adherence | Type IV pili |
| WP_149397158.1, WP_149399025.1 | 19.22 - 20.46 | VFG002436 | Adherence | BoaA |
| WP_149397180.1 | 21.86 | VFG034541 | Adherence | EtpA |
| WP_149397205.1, WP_223146182.1 | 23.78 - 27.11 | VFG050685 | Adherence | TFP |
| WP_149397470.1 | 31.96 | VFG004125 | Adherence | Agf |
| WP_149397744.1 | 28.28 | VFG001931 | Adherence | CadF |
| WP_149397761.1 | 26.66 | VFG050303 | Adherence | Ata |
| WP_149397763.1 | 36.13 | VFG050640 | Adherence | TFP |
| WP_149398594.1 | 24.6 | VFG038505 | Adherence | Tap type IV pili |
| WP_149398658.1 | 30.49 | VFG000872 | Adherence | Type 1 fimbriae |
| WP_149398733.1 | 39.07 | VFG002428 | Adherence | Type IV pili |
| WP_149398745.1 | 38.56 | VFG045346 | Adherence | IlpA |
| WP_149398760.1 | 23.34 | VFG038444 | Adherence | Tap type IV pili |
| WP_255474880.1 | 26.63 | VFG000098 | Adherence | TCP |
| WP_255474881.1 | 38.27 | VFG042966 | Adherence | Type IV pili |
| WP_255474968.1 | 27.66 | VFG001713 | Adherence | P fimbriae |
| WP_255475068.1 | 34.18 | VFG042569 | Adherence | F1C fimbriae |
| WP_149395257.1, WP_149395497.1, WP_149395726.1, WP_149396427.1, WP_149397294.1, WP_255474994.1 | 22.07 - 30.2 | VFG036956 | Antimicrobial activity/Competitive advantage | FarAB |
| WP_149395776.1 | 22.32 | VFG036938 | Antimicrobial activity/Competitive advantage | FarAB |
| WP_149396457.1, WP_149398409.1 | 21.39 - 29.15 | VFG002301 | Antimicrobial activity/Competitive advantage | Mig-5 |
| WP_149399100.1 | 21.21 | VFG036992 | Antimicrobial activity/Competitive advantage | MtrCDE |
| WP_149395471.1, WP_149395475.1, WP_149396072.1, WP_149399528.1, WP_188074504.1 | 20.7 - 26.41 | VFG002197 | Biofilm | BopD |
| WP_149395617.1, WP_149397843.1, WP_149398816.1, WP_149399098.1, WP_188074634.1, WP_255474949.1 | 22.54 - 29.34 | VFG037735 | Biofilm | AdeFGH efflux pump |
| WP_149396143.1 | 25.58 | VFG050279 | Biofilm | VPS |
| WP_149396403.1, WP_188074628.1 | 28.26 - 29.82 | VFG037612 | Biofilm | Csu fimbriae |
| WP_149396404.1, WP_149398245.1 | 27.06 - 31.67 | VFG037599 | Biofilm | Csu fimbriae |
| WP_149396929.1 | 35.14 | VFG050274 | Biofilm | VPS |
| WP_149397050.1 | 21.93 | VFG050280 | Biofilm | VPS |
| WP_149397147.1 | 42.49 | VFG000130 | Biofilm | Alginate |
| WP_149397148.1 | 30.28 | VFG000131 | Biofilm | Alginate |
| WP_149397188.1, WP_149398920.1, WP_149399770.1 | 26.55 - 61.26 | VFG000121 | Biofilm | Alginate |
| WP_149397335.1, WP_149398818.1, WP_188074670.1 | 21.71 - 26.63 | VFG037705 | Biofilm | AdeFGH efflux pump |
| WP_149397577.1 | 71.65 | VFG037720 | Biofilm | AdeFGH efflux pump |
| WP_149397937.1 | 31.07 | VFG050281 | Biofilm | VPS |
| WP_149398243.1 | 30.15 | VFG037625 | Biofilm | Csu fimbriae |
| WP_149398635.1 | 24.67 | VFG014893 | Biofilm | Alginate |
| WP_149398986.1 | 40.79 | VFG000116 | Biofilm | Alginate |
| WP_149399327.1 | 55.66 | VFG014984 | Biofilm | Alginate |
| WP_149399522.1 | 24.31 | VFG037664 | Biofilm | PNAG |
| WP_149399767.1 | 41.65 | VFG014950 | Biofilm | Alginate |
| WP_255474926.1 | 41.3 | VFG001284 | Biofilm | Intercellular adhesion proteins |
| WP_255475103.1 | 27.97 | VFG000135 | Biofilm | Alginate |
| WP_149395303.1 | 47.17 | VFG002440 | Effector delivery system | Bsa T3SS |
| WP_149395630.1 | 26.28 | VFG051235 | Effector delivery system | T4SS secreted effectors |
| WP_149395633.1 | 50.43 | VFG041031 | Effector delivery system | HSI-3 |
| WP_149395742.1, WP_149395963.1, WP_149397208.1 | 29.17 - 29.67 | VFG041423 | Effector delivery system | T4SS secreted effectors |
| WP_149395783.1, WP_149398248.1, WP_149398966.1, WP_188074523.1 | 26.04 - 33.99 | VFG000493 | Effector delivery system | TTSS (SPI-2 encode) |
| WP_149395802.1 | 42.11 | VFG039549 | Effector delivery system | T4SS secreted effectors |
| WP_149395912.1, WP_149396037.1 | 23.42 - 33.33 | VFG000492 | Effector delivery system | TTSS (SPI-2 encode) |
| WP_149395985.1, WP_149395986.1, WP_149396901.1, WP_149397901.1 | 26.13 - 33.22 | VFG050222 | Effector delivery system | TTSS secreted effectors |
| WP_149396005.1, WP_149398946.1 | 26.92 - 29.29 | VFG039623 | Effector delivery system | T4SS secreted effectors |
| WP_149396015.1 | 21.3 | VFG002099 | Effector delivery system | Dot/Icm T4SS secreted effectors |
| WP_149396253.1, WP_149397995.1, WP_255474965.1 | 25.12 - 29.71 | VFG039696 | Effector delivery system | T4SS secreted effectors |
| WP_149396707.1 | 29.32 | VFG000515 | Effector delivery system | TTSS (SPI-2 encode) |
| WP_149396814.1 | 51.87 | VFG039536 | Effector delivery system | T4SS secreted effectors |
| WP_149397008.1 | 38.3 | VFG002208 | Effector delivery system | VirB type IV secretion system |
| WP_149397159.1, WP_149398607.1 | 26.67 - 32.22 | VFG007177 | Effector delivery system | T3SS1 |
| WP_149397204.1, WP_149398613.1 | 36.14 - 36.68 | VFG039430 | Effector delivery system | T4SS secreted effectors |
| WP_149397230.1, WP_149399520.1 | 27.98 - 30 | VFG045590 | Effector delivery system | Dot/Icm T4SS secreted effectors |
| WP_149397324.1 | 35.08 | VFG000390 | Effector delivery system | TTSS |
| WP_149397330.1 | 28.38 | VFG041431 | Effector delivery system | T4SS secreted effectors |
| WP_149397333.1 | 37.09 | VFG002059 | Effector delivery system | HSI-1 |
| WP_149397444.1 | 18.08 | VFG051244 | Effector delivery system | T4SS secreted effectors |
| WP_149398034.1, WP_149399230.1 | 37.74 - 50 | VFG045340 | Effector delivery system | T4SS secreted effectors |
| WP_149398056.1 | 30.41 | VFG045567 | Effector delivery system | Dot/Icm T4SS secreted effectors |
| WP_149398143.1 | 50.82 | VFG050255 | Effector delivery system | TTSS secreted effectors |
| WP_149398168.1 | 26.37 | VFG002244 | Effector delivery system | VirB/VirD4 T4SS secreted effectors |
| WP_149398292.1 | 36.28 | VFG049649 | Effector delivery system | TTSS secreted effectors |
| WP_149398414.1 | 30.07 | VFG002409 | Effector delivery system | Type VII secretion system |
| WP_149398699.1 | 28.51 | VFG045607 | Effector delivery system | Dot/Icm T4SS secreted effectors |
| WP_149398836.1 | 38.41 | VFG045476 | Effector delivery system | Dot/Icm T4SS secreted effectors |
| WP_149398998.1 | 32.63 | VFG000537 | Effector delivery system | TTSS (SPI-1 encode) |
| WP_149399007.1 | 28.77 | VFG000048 | Effector delivery system | TTSS |
| WP_149399009.1 | 27.69 | VFG000192 | Effector delivery system | TTSS |
| WP_149399493.1 | 37.91 | VFG039615 | Effector delivery system | T4SS secreted effectors |
| WP_149399652.1 | 32.91 | VFG039491 | Effector delivery system | T4SS secreted effectors |
| WP_149399857.1 | 40.58 | VFG039386 | Effector delivery system | T4SS secreted effectors |
| WP_149399858.1 | 40.74 | VFG041119 | Effector delivery system | T6SS-1 |
| WP_188074488.1 | 31.56 | VFG041321 | Effector delivery system | Dot/Icm T4SS secreted effectors |
| WP_255474802.1 | 34.83 | VFG000539 | Effector delivery system | TTSS (SPI-1 encode) |
| WP_255474805.1 | 21.78 | VFG042196 | Effector delivery system | TTSS secreted effectors |
| WP_255474892.1 | 36 | VFG041304 | Effector delivery system | Dot/Icm T4SS secreted effectors |
| WP_255474910.1 | 40.41 | VFG039371 | Effector delivery system | T4SS secreted effectors |
| WP_255474963.1 | 48.71 | VFG042069 | Effector delivery system | TTSS |
| WP_255475001.1 | 34.74 | VFG010515 | Effector delivery system | Dot/Icm T4SS secreted effectors |
| WP_149395418.1, WP_149395539.1, WP_149396048.1, WP_149396647.1, WP_149398604.1, WP_149398720.1 | 23.58 - 35.02 | VFG040705 | Exoenzyme | TlyC |
| WP_149395268.1 | 24.47 | VFG005784 | Exotoxin | <beta>-haemolysin/cytolysin |
| WP_149395333.1, WP_149395835.1, WP_149397432.1, WP_149397433.1, WP_149397663.1, WP_149397806.1, WP_149397894.1, WP_149398387.1, WP_223146300.1 | 27.57 - 34.93 | VFG005775 | Exotoxin | <beta>-haemolysin/cytolysin |
| WP_149395344.1, WP_149395465.1, WP_149396168.1, WP_149397516.1, WP_149398219.1, WP_149399284.1 | 21.2 - 32.06 | VFG049150 | Exotoxin | Colibactin |
| WP_149395391.1, WP_149397337.1, WP_149397573.1, WP_149398120.1 | 19.3 - 37.71 | VFG037203 | Exotoxin | Phospholipase D |
| WP_149395404.1, WP_149397166.1, WP_149397222.1, WP_149397493.1, WP_149397639.1, WP_149398467.1, WP_149398891.1, WP_149399070.1, WP_149399370.1, WP_188074507.1, WP_188074561.1, WP_188074584.1, WP_255474838.1 | 23.08 - 36.13 | VFG049158 | Exotoxin | Colibactin |
| WP_149395456.1, WP_149398254.1, WP_149399195.1, WP_149399825.1 | 23.48 - 32.91 | VFG000907 | Exotoxin | <alpha>-Hemolysin |
| WP_149395537.1, WP_149395538.1, WP_149395621.1, WP_149395724.1, WP_149395813.1, WP_149395952.1, WP_149396169.1, WP_149396170.1, WP_149396735.1, WP_149397047.1, WP_149397061.1, WP_149398001.1, WP_149398084.1, WP_149398098.1, WP_149398221.1, WP_149398308.1, WP_149398394.1, WP_149398561.1, WP_149398570.1, WP_149398687.1, WP_149398934.1, WP_149398935.1, WP_149399051.1, WP_255474972.1 | 22.63 - 32.12 | VFG049152 | Exotoxin | Colibactin |
| WP_149396115.1, WP_149397822.1, WP_149398819.1 | 24.45 - 26.63 | VFG001827 | Exotoxin | ShET2 |
| WP_149396458.1, WP_149397396.1 | 25.44 - 25.78 | VFG049162 | Exotoxin | Colibactin |
| WP_149396561.1 | 27.12 | VFG000841 | Exotoxin | Hemolysin |
| WP_149397660.1, WP_149398725.1 | 26.13 - 27.8 | VFG038916 | Exotoxin | RtxA |
| WP_149397716.1, WP_149398622.1 | 22.99 - 27.75 | VFG050113 | Exotoxin | Cereulide |
| WP_149397746.1 | 21.43 | VFG050118 | Exotoxin | Cereulide |
| WP_149397817.1 | 29.46 | VFG005766 | Exotoxin | <beta>-haemolysin/cytolysin |
| WP_149398483.1 | 28.78 | VFG050117 | Exotoxin | Cereulide |
| WP_149398609.1 | 28.39 | VFG002176 | Exotoxin | Cytolysin |
| WP_149399475.1 | 23.95 | VFG049148 | Exotoxin | Colibactin |
| WP_013742768.1 | 45.45 | VFG011430 | Immune modulation | LPS |
| WP_149395282.1 | 61.42 | VFG000430 | Immune modulation | Vi antigen |
| WP_149395323.1, WP_149396811.1, WP_149397237.1, WP_149397562.1, WP_149398119.1, WP_149399920.1 | 20.37 - 29.97 | VFG007849 | Immune modulation | PDIM |
| WP_149395437.1 | 23.89 | VFG002361 | Immune modulation | O-antigen |
| WP_149395445.1, WP_149395844.1, WP_149397364.1, WP_149397735.1 | 23.38 - 30.5 | VFG048830 | Immune modulation | Capsule |
| WP_149395775.1, WP_149397173.1, WP_149397861.1, WP_149397972.1, WP_149398008.1, WP_149398009.1 | 28.87 - 33.93 | VFG007904 | Immune modulation | PDIM |
| WP_149395825.1 | 30.66 | VFG000307 | Immune modulation | HP-NAP |
| WP_149395994.1 | 25.52 | VFG052332 | Immune modulation | LPS |
| WP_149396031.1 | 24.78 | VFG046955 | Immune modulation | LPS |
| WP_149396076.1 | 25.71 | VFG002028 | Immune modulation | LOS |
| WP_149396161.1 | 25.09 | VFG052324 | Immune modulation | LPS |
| WP_149396186.1 | 29.29 | VFG033025 | Immune modulation | PgdA |
| WP_149396213.1 | 66.3 | VFG013465 | Immune modulation | LOS |
| WP_149396263.1 | 37.77 | VFG038091 | Immune modulation | Capsule |
| WP_149396301.1 | 23.45 | VFG001970 | Immune modulation | Capsule |
| WP_149396307.1, WP_149396742.1, WP_149397844.1 | 36.62 - 45.39 | VFG000331 | Immune modulation | LOS |
| WP_149396317.1 | 38.89 | VFG002548 | Immune modulation | Capsule I |
| WP_149396319.1 | 40.66 | VFG002549 | Immune modulation | Capsule I |
| WP_149396320.1, WP_149398643.1 | 25.45 - 37.5 | VFG002550 | Immune modulation | Capsule I |
| WP_149396566.1, WP_149399516.1 | 36.99 - 37.29 | VFG013248 | Immune modulation | LOS |
| WP_149396608.1, WP_149398082.1 | 23.53 - 33.09 | VFG000028 | Immune modulation | Brk |
| WP_149396609.1 | 26.67 | VFG013476 | Immune modulation | LOS |
| WP_149396682.1, WP_149396750.1, WP_149398704.1 | 26.53 - 34.29 | VFG001965 | Immune modulation | Capsule |
| WP_149396755.1 | 34.21 | VFG002552 | Immune modulation | Capsule I |
| WP_149396764.1 | 23.77 | VFG037777 | Immune modulation | LPS |
| WP_149396829.1 | 35 | VFG013496 | Immune modulation | LOS |
| WP_149396839.1, WP_149397233.1, WP_149397454.1, WP_223146278.1 | 23.34 - 29.88 | VFG001408 | Immune modulation | PDIM |
| WP_149396859.1 | 23.49 | VFG014100 | Immune modulation | LPS |
| WP_149397247.1 | 28.37 | VFG000696 | Immune modulation | Capsule |
| WP_149397796.1 | 48.95 | VFG000320 | Immune modulation | LPS |
| WP_149397889.1 | 29.45 | VFG002230 | Immune modulation | LPS |
| WP_149397936.1 | 47.15 | VFG047256 | Immune modulation | LPS |
| WP_149397953.1, WP_149399778.1 | 35.14 - 35.58 | VFG024730 | Immune modulation | PDIM |
| WP_149398018.1 | 26.79 | VFG008007 | Immune modulation | Sulfolipid-1 biosynthesis and transport |
| WP_149398051.1 | 24.1 | VFG001310 | Immune modulation | Capsule |
| WP_149398169.1 | 28.68 | VFG002226 | Immune modulation | LPS |
| WP_149398490.1 | 42.31 | VFG002219 | Immune modulation | C<beta>G |
| WP_149398494.1 | 34.46 | VFG002546 | Immune modulation | Capsule I |
| WP_149398673.1 | 23.12 | VFG001963 | Immune modulation | Capsule |
| WP_149398683.1 | 35.42 | VFG011823 | Immune modulation | LOS |
| WP_149398758.1 | 47.51 | VFG013412 | Immune modulation | LOS |
| WP_149398767.1 | 22.53 | VFG045310 | Immune modulation | LPS |
| WP_149398866.1 | 27.25 | VFG024748 | Immune modulation | PDIM |
| WP_149399313.1, WP_149399315.1 | 30.6 - 43.08 | VFG045297 | Immune modulation | LPS |
| WP_149399518.1 | 24.1 | VFG013400 | Immune modulation | LOS |
| WP_149399774.1 | 28.83 | VFG007832 | Immune modulation | PDIM |
| WP_149399922.1 | 28.83 | VFG007965 | Immune modulation | PDIM |
| WP_188074505.1 | 26.14 | VFG014105 | Immune modulation | LPS |
| WP_188074542.1 | 24.32 | VFG014113 | Immune modulation | LPS |
| WP_188074673.1 | 30.18 | VFG000137 | Immune modulation | LPS |
| WP_188074674.1 | 29.92 | VFG046628 | Immune modulation | Capsule |
| WP_223146193.1 | 30.54 | VFG047176 | Immune modulation | LPS |
| WP_255474763.1 | 26.33 | VFG049040 | Immune modulation | LPS |
| WP_149395777.1 | 36.08 | VFG034679 | Invasion | Ibes |
| WP_149396297.1 | 35.96 | VFG050163 | Invasion | K1 capsule |
| WP_149395250.1, WP_149395467.1, WP_149395533.1, WP_149397375.1, WP_149397397.1, WP_149397553.1, WP_149397554.1, WP_149397617.1, WP_149398398.1, WP_149398868.1, WP_149399776.1, WP_149399904.1, WP_223146289.1, WP_255474952.1 | 23.2 - 54.39 | VFG038840 | Motility | Polar flagella |
| WP_149397141.1 | 24.8 | VFG043350 | Motility | Flagella |
| WP_149397443.1 | 32.11 | VFG014720 | Motility | Flagella |
| WP_149397494.1 | 31.02 | VFG014369 | Motility | Flagella |
| WP_149397678.1 | 35.76 | VFG007454 | Motility | Flagella |
| WP_149397904.1, WP_149399717.1 | 43.02 - 52.61 | VFG001248 | Motility | Flagella |
| WP_149398173.1 | 29.07 | VFG038723 | Motility | Polar flagella |
| WP_149398324.1 | 26.94 | VFG001895 | Motility | Flagella |
| WP_149398335.1, WP_149398433.1 | 24.03 - 36.14 | VFG007490 | Motility | Flagella |
| WP_149398548.1, WP_223146269.1 | 25.24 - 30.6 | VFG011850 | Motility | Flagella |
| WP_255474872.1 | 26.52 | VFG011938 | Motility | Flagella |
| WP_137238322.1, WP_149395767.1 | 36.54 - 55.58 | VFG001814 | Nutritional/Metabolic factor | Nitrate reductase |
| WP_149395286.1, WP_149395486.1, WP_149397340.1, WP_149398616.1, WP_149399053.1, WP_188074666.1, WP_255474935.1 | 20.86 - 27.6 | VFG037470 | Nutritional/Metabolic factor | HemO cluster |
| WP_149395292.1, WP_149395414.1, WP_149395723.1, WP_149396096.1, WP_149396335.1, WP_149396565.1, WP_149396603.1, WP_149396822.1, WP_149396918.1, WP_149397372.1, WP_149397649.1, WP_149397789.1, WP_149398013.1, WP_149398102.1, WP_149398200.1, WP_149398517.1, WP_149398937.1, WP_149399386.1, WP_188074619.1 | 19.92 - 31.6 | VFG049118 | Nutritional/Metabolic factor | Allantion utilization |
| WP_149395301.1, WP_149395472.1, WP_149395865.1, WP_149396069.1, WP_149396106.1, WP_149396340.1, WP_149396371.1, WP_149396665.1, WP_149397114.1, WP_149397153.1, WP_149397154.1, WP_149397431.1, WP_149397455.1, WP_149397807.1, WP_149398045.1, WP_149398090.1, WP_149398261.1, WP_149399094.1, WP_255475014.1 | 27.19 - 40.87 | VFG001206 | Nutritional/Metabolic factor | FbpABC |
| WP_149395314.1 | 25 | VFG000934 | Nutritional/Metabolic factor | Enterobactin |
| WP_149395366.1, WP_149395431.1, WP_149395483.1, WP_149395623.1, WP_149396238.1, WP_149396280.1, WP_149396631.1, WP_149396660.1, WP_149396766.1, WP_149397075.1, WP_149397220.1, WP_149397285.1, WP_149397293.1, WP_149397295.1, WP_149397352.1, WP_149397466.1, WP_149397487.1, WP_149397579.1, WP_149397586.1, WP_149397922.1, WP_149397954.1, WP_149398081.1, WP_149398127.1, WP_149398944.1, WP_149399091.1, WP_149399372.1, WP_188074569.1, WP_223146236.1, WP_255474962.1, WP_255475080.1 | 22.18 - 36.46 | VFG044083 | Nutritional/Metabolic factor | Pyoverdine |
| WP_149395371.1, WP_149395620.1, WP_149395814.1, WP_149396217.1, WP_149396255.1, WP_149396510.1, WP_149397072.1, WP_149397096.1, WP_149397125.1, WP_149397212.1, WP_149398553.1, WP_149398995.1 | 24 - 43.55 | VFG049114 | Nutritional/Metabolic factor | Allantion utilization |
| WP_149395373.1, WP_149396530.1, WP_149397115.1, WP_149397420.1, WP_149398129.1, WP_149398864.1, WP_149399188.1 | 25.78 - 30.65 | VFG016046 | Nutritional/Metabolic factor | Pyoverdine |
| WP_149395464.1, WP_149395999.1, WP_149397349.1, WP_149398411.1 | 28.17 - 33.18 | VFG049994 | Nutritional/Metabolic factor | Bacillibactin |
| WP_149395490.1, WP_149397948.1, WP_255474873.1 | 26.26 - 28.11 | VFG001817 | Nutritional/Metabolic factor | Mycobactin |
| WP_149395503.1 | 24.5 | VFG016041 | Nutritional/Metabolic factor | Pyoverdine |
| WP_149395536.1 | 26.76 | VFG044332 | Nutritional/Metabolic factor | Ybt |
| WP_149395738.1, WP_149397575.1, WP_149397775.1, WP_149398242.1 | 28.5 - 42.03 | VFG044082 | Nutritional/Metabolic factor | Pyoverdine |
| WP_149395782.1, WP_149398215.1, WP_149398216.1, WP_149398932.1, WP_149399108.1, WP_149399270.1, WP_188074575.1, WP_255474778.1, WP_255474941.1, WP_255474973.1 | 23.2 - 28.14 | VFG050016 | Nutritional/Metabolic factor | Bacillibactin |
| WP_149395816.1, WP_149396496.1 | 23.08 - 29.24 | VFG000919 | Nutritional/Metabolic factor | Chu |
| WP_149395860.1, WP_149396098.1, WP_149396233.1, WP_149398391.1 | 25.09 - 27.57 | VFG000168 | Nutritional/Metabolic factor | Pyochelin |
| WP_149395898.1, WP_149398559.1 | 30.1 - 33.54 | VFG000925 | Nutritional/Metabolic factor | Enterobactin |
| WP_149395973.1, WP_149396060.1, WP_149398060.1, WP_149398996.1, WP_223146264.1, WP_223146298.1 | 20.82 - 49.26 | VFG000574 | Nutritional/Metabolic factor | MgtBC |
| WP_149395988.1 | 40 | VFG001416 | Nutritional/Metabolic factor | PanC/PanD |
| WP_149396081.1, WP_149396431.1, WP_149396799.1, WP_149396914.1, WP_149397279.1, WP_149397346.1, WP_149397424.1, WP_149398037.1, WP_149398038.1, WP_149398149.1, WP_149398600.1, WP_149398610.1, WP_149398747.1, WP_149398796.1, WP_149398880.1, WP_149398911.1, WP_149398921.1, WP_188074622.1, WP_223146256.1, WP_255474897.1, WP_255474942.1 | 25.1 - 41.72 | VFG000344 | Nutritional/Metabolic factor | HitABC |
| WP_149396111.1, WP_149397332.1 | 21.56 - 30.4 | VFG044097 | Nutritional/Metabolic factor | Pyochelin |
| WP_149396116.1 | 28.48 | VFG044334 | Nutritional/Metabolic factor | Sal |
| WP_149396246.1, WP_149396528.1, WP_149398568.1, WP_149399115.1 | 26.23 - 35.69 | VFG044084 | Nutritional/Metabolic factor | Pyoverdine |
| WP_149396248.1 | 27.32 | VFG050027 | Nutritional/Metabolic factor | Bacillibactin |
| WP_149396353.1 | 27.57 | VFG037260 | Nutritional/Metabolic factor | Acinetobactin |
| WP_149396359.1 | 26.68 | VFG049962 | Nutritional/Metabolic factor | Petrobactin |
| WP_149396361.1 | 25.25 | VFG037358 | Nutritional/Metabolic factor | Acinetobactin |
| WP_149396394.1, WP_149396637.1 | 33.81 - 38.73 | VFG047690 | Nutritional/Metabolic factor | Pyrimidine biosynthesis |
| WP_149396518.1, WP_149397641.1, WP_149399055.1 | 22.83 - 30.18 | VFG000932 | Nutritional/Metabolic factor | Enterobactin |
| WP_149396524.1 | 41.25 | VFG037482 | Nutritional/Metabolic factor | HemO cluster |
| WP_149396526.1, WP_149398352.1, WP_149398820.1, WP_149398952.1 | 21.12 - 36.17 | VFG000163 | Nutritional/Metabolic factor | Pyoverdine |
| WP_149396559.1 | 21.55 | VFG015994 | Nutritional/Metabolic factor | Pyoverdine |
| WP_149396563.1, WP_149397540.1 | 19.21 - 25.98 | VFG002097 | Nutritional/Metabolic factor | Pht |
| WP_149396768.1 | 28.63 | VFG012509 | Nutritional/Metabolic factor | Salmochelin siderophore |
| WP_149396769.1, WP_149397897.1, WP_149398556.1, WP_149398772.1, WP_149398989.1 | 24.4 - 28.74 | VFG037386 | Nutritional/Metabolic factor | Acinetobactin |
| WP_149396815.1 | 32.27 | VFG047582 | Nutritional/Metabolic factor | Purine biosynthesis |
| WP_149396911.1 | 27.07 | VFG047726 | Nutritional/Metabolic factor | Pyrimidine biosynthesis |
| WP_149396967.1 | 36.78 | VFG045330 | Nutritional/Metabolic factor | Legiobactin |
| WP_149397110.1, WP_188074461.1 | 24.31 - 26.06 | VFG047529 | Nutritional/Metabolic factor | Biotin synthesis |
| WP_149397134.1 | 38.64 | VFG049120 | Nutritional/Metabolic factor | Allantion utilization |
| WP_149397353.1 | 34.44 | VFG015512 | Nutritional/Metabolic factor | Pyocyanin |
| WP_149397421.1, WP_149398617.1, WP_149398790.1, WP_223146250.1 | 27.14 - 35.19 | VFG047600 | Nutritional/Metabolic factor | GGT |
| WP_149397594.1, WP_149398922.1, WP_149399096.1, WP_149399534.1, WP_255475050.1 | 19.04 - 25 | VFG000249 | Nutritional/Metabolic factor | FbpABC |
| WP_149397698.1 | 29.19 | VFG002158 | Nutritional/Metabolic factor | LplA1 |
| WP_149397770.1 | 28.03 | VFG047672 | Nutritional/Metabolic factor | ArgP |
| WP_149397791.1 | 34.89 | VFG048488 | Nutritional/Metabolic factor | Ent |
| WP_149397816.1, WP_149397917.1, WP_188074529.1 | 21.71 - 24.22 | VFG000164 | Nutritional/Metabolic factor | Pyochelin |
| WP_149397839.1 | 26.73 | VFG048547 | Nutritional/Metabolic factor | Sal |
| WP_149397840.1, WP_149398777.1 | 20.97 - 36.36 | VFG013065 | Nutritional/Metabolic factor | Shu |
| WP_149397941.1, WP_149397974.1 | 25.67 - 28.62 | VFG049124 | Nutritional/Metabolic factor | Allantion utilization |
| WP_149398121.1 | 34.52 | VFG047546 | Nutritional/Metabolic factor | Biotin synthesis |
| WP_149398134.1 | 23.38 | VFG012597 | Nutritional/Metabolic factor | Chu |
| WP_149398148.1 | 29.8 | VFG013070 | Nutritional/Metabolic factor | Shu |
| WP_149398255.1 | 20.54 | VFG010858 | Nutritional/Metabolic factor | Legiobactin |
| WP_149398339.1, WP_149398608.1 | 22.95 - 23.05 | VFG050038 | Nutritional/Metabolic factor | Bacillibactin |
| WP_149398368.1 | 26.07 | VFG044197 | Nutritional/Metabolic factor | Rhizoferrin |
| WP_149398498.1 | 25.66 | VFG000161 | Nutritional/Metabolic factor | Pyoverdine |
| WP_149398564.1 | 32.28 | VFG015542 | Nutritional/Metabolic factor | Pyocyanin |
| WP_149398821.1, WP_149399354.1 | 29.25 - 31.79 | VFG044087 | Nutritional/Metabolic factor | Pyoverdine |
| WP_149398822.1 | 25.83 | VFG044086 | Nutritional/Metabolic factor | Pyoverdine |
| WP_149398849.1 | 21.91 | VFG047708 | Nutritional/Metabolic factor | Pyrimidine biosynthesis |
| WP_149398904.1 | 24.15 | VFG044096 | Nutritional/Metabolic factor | Pyochelin |
| WP_149399135.1 | 28.85 | VFG009561 | Nutritional/Metabolic factor | Mycobactin |
| WP_149399137.1 | 28.12 | VFG015515 | Nutritional/Metabolic factor | Pyocyanin |
| WP_149399205.1 | 27.38 | VFG016005 | Nutritional/Metabolic factor | Pyoverdine |
| WP_149399217.1 | 35.45 | VFG047431 | Nutritional/Metabolic factor | Biotin synthesis |
| WP_188074581.1, WP_188074686.1, WP_255474869.1 | 19.64 - 32.68 | VFG037513 | Nutritional/Metabolic factor | HemO cluster |
| WP_188074582.1 | 28.79 | VFG000162 | Nutritional/Metabolic factor | Pyoverdine |
| WP_188074646.1 | 25.88 | VFG016058 | Nutritional/Metabolic factor | Pyoverdine |
| WP_188074685.1 | 24.77 | VFG000339 | Nutritional/Metabolic factor | Tbp |
| WP_223146201.1 | 29.82 | VFG000367 | Nutritional/Metabolic factor | Yersiniabactin |
| WP_255474816.1 | 27.11 | VFG044322 | Nutritional/Metabolic factor | Sal |
| WP_255474837.1 | 27.66 | VFG000343 | Nutritional/Metabolic factor | HitABC |
| WP_255474850.1 | 31.95 | VFG037344 | Nutritional/Metabolic factor | Acinetobactin |
| WP_255474851.1 | 23.35 | VFG000616 | Nutritional/Metabolic factor | Aerobactin |
| WP_255474919.1 | 22.26 | VFG000917 | Nutritional/Metabolic factor | Chu |
| WP_255474945.1 | 29.92 | VFG037497 | Nutritional/Metabolic factor | HemO cluster |
| WP_255474974.1 | 23.9 | VFG000268 | Nutritional/Metabolic factor | HpuAB |
| WP_255475062.1 | 49.84 | VFG047512 | Nutritional/Metabolic factor | Biotin synthesis |
| WP_149397984.1 | 30.25 | VFG001385 | Others | MmaA4 |
| WP_149398883.1, WP_255475063.1 | 30.41 - 34.76 | VFG035923 | Others | Dispersin |
| WP_149399719.1 | 22.33 | VFG001381 | Others | Isocitrate lyase |
| WP_149395255.1, WP_149396114.1, WP_149397736.1, WP_149398504.1, WP_149398961.1, WP_255474867.1 | 27.92 - 30.65 | VFG052339 | Regulation | PmrAB |
| WP_149395256.1, WP_149398190.1 | 29.95 - 31.11 | VFG001390 | Regulation | MprAB |
| WP_149395792.1, WP_149397149.1 | 26.9 - 32.2 | VFG045467 | Regulation | CdpA |
| WP_149395891.1 | 35.49 | VFG001826 | Regulation | RelA |
| WP_149396113.1, WP_149397887.1 | 28.36 - 32.11 | VFG000475 | Regulation | PhoPQ |
| WP_149397206.1 | 28.71 | VFG002044 | Regulation | BvgAS |
| WP_149397286.1, WP_149397542.1, WP_149398155.1, WP_149398188.1 | 26.79 - 30.04 | VFG009810 | Regulation | PhoP |
| WP_149397737.1 | 34.4 | VFG052340 | Regulation | PmrAB |
| WP_149397918.1 | 28.36 | VFG001888 | Regulation | LetA/S |
| WP_149397923.1 | 31.36 | VFG000477 | Regulation | RpoS |
| WP_149398107.1, WP_149398500.1, WP_149398744.1 | 24.23 - 27.24 | VFG002045 | Regulation | BvgAS |
| WP_188074525.1 | 24.31 | VFG001392 | Regulation | SigE |
| WP_188074641.1, WP_255475104.1 | 27.92 - 28.29 | VFG009869 | Regulation | MprAB |
| WP_255474966.1 | 23.81 | VFG001825 | Regulation | DevRS |
| WP_149395756.1, WP_149395757.1 | 20.72 - 23.75 | VFG037064 | Stress survival | MntABC |
| WP_149395758.1 | 31.46 | VFG037082 | Stress survival | MntABC |
| WP_149396332.1 | 25.14 | VFG000080 | Stress survival | ClpE |
| ANT_WB102 | | | | |
| WP_149290832.1, WP_149290833.1 | 35.25 - 37.99 | VFG001811 | Adherence | Antigen 85 |
| WP_149291549.1 | 36.93 | VFG001383 | Adherence | HbhA |
| WP_149291802.1 | 46.43 | VFG000449 | Adherence | Type 1 fimbriae |
| WP_149291817.1, WP_149291936.1, WP_149292275.1 | 31.17 - 31.39 | VFG046465 | Adherence | EF-Tu |
| WP_149292891.1 | 33.45 | VFG042739 | Adherence | Type IV pili |
| WP_149293143.1 | 58.41 | VFG050291 | Adherence | DIP1621 |
| WP_188065019.1 | 50.66 | VFG050289 | Adherence | DIP0733 |
| WP_255583285.1 | 38.87 | VFG043634 | Adherence | Flp pili |
| WP_255584007.1 | 33.33 | VFG050290 | Adherence | DIP1281 |
| WP_149290636.1, WP_149293051.1 | 31.32 - 33.5 | VFG002301 | Antimicrobial activity/Competitive advantage | Mig-5 |
| WP_149292143.1 | 30 | VFG036956 | Antimicrobial activity/Competitive advantage | FarAB |
| WP_149290672.1 | 34.07 | VFG037678 | Biofilm | PNAG |
| WP_149291281.1 | 31.34 | VFG050279 | Biofilm | VPS |
| WP_149293338.1 | 34.62 | VFG002540 | Biofilm | Quorum-sensing |
| WP_255583921.1 | 40.88 | VFG014984 | Biofilm | Alginate |
| WP_149290837.1, WP_149290971.1, WP_149292339.1 | 31.35 - 45.35 | VFG041304 | Effector delivery system | Dot/Icm T4SS secreted effectors |
| WP_149290962.1 | 35.11 | VFG002061 | Effector delivery system | HSI-1 |
| WP_149290965.1, WP_149290966.1, WP_255584364.1 | 35.11 - 37.84 | VFG002060 | Effector delivery system | HSI-1 |
| WP_149290994.1 | 37.68 | VFG050255 | Effector delivery system | TTSS secreted effectors |
| WP_149291157.1, WP_149291733.1 | 37.39 - 38.34 | VFG002059 | Effector delivery system | HSI-1 |
| WP_149291380.1 | 31.43 | VFG003997 | Effector delivery system | TTSS-2 secreted effectors |
| WP_149292222.1 | 40.85 | VFG045340 | Effector delivery system | T4SS secreted effectors |
| WP_149292332.1 | 44.53 | VFG039536 | Effector delivery system | T4SS secreted effectors |
| WP_149292400.1 | 41.27 | VFG041318 | Effector delivery system | Dot/Icm T4SS secreted effectors |
| WP_149293105.1 | 38.14 | VFG039691 | Effector delivery system | T4SS secreted effectors |
| WP_149293192.1, WP_255584368.1 | 30.65 - 39.2 | VFG050222 | Effector delivery system | TTSS secreted effectors |
| WP_255584094.1 | 33.71 | VFG041423 | Effector delivery system | T4SS secreted effectors |
| WP_149290685.1, WP_149292937.1 | 30.4 - 31.18 | VFG049152 | Exotoxin | Colibactin |
| WP_149291263.1, WP_149291462.1 | 30.77 - 32.27 | VFG005766 | Exotoxin | <beta>-haemolysin/cytolysin |
| WP_149291544.1, WP_149292818.1 | 32.27 - 34.53 | VFG049150 | Exotoxin | Colibactin |
| WP_149291692.1 | 30.85 | VFG049158 | Exotoxin | Colibactin |
| WP_149293093.1 | 40.47 | VFG050113 | Exotoxin | Cereulide |
| WP_149293254.1 | 31.07 | VFG000907 | Exotoxin | <alpha>-Hemolysin |
| WP_095719155.1 | 45.45 | VFG011430 | Immune modulation | LPS |
| WP_149290677.1 | 31.63 | VFG047149 | Immune modulation | LPS |
| WP_149290808.1 | 31.05 | VFG007849 | Immune modulation | PDIM |
| WP_149290814.1 | 42.48 | VFG002228 | Immune modulation | LPS |
| WP_149290825.1 | 41.92 | VFG052045 | Immune modulation | LAM |
| WP_149290828.1 | 38.6 | VFG007823 | Immune modulation | PDIM |
| WP_149290829.1 | 36.39 | VFG001407 | Immune modulation | PDIM |
| WP_149290839.1 | 44.09 | VFG002182 | Immune modulation | Capsule |
| WP_149290884.1, WP_149291564.1 | 31.64 - 32.74 | VFG024748 | Immune modulation | PDIM |
| WP_149291008.1 | 36.1 | VFG052155 | Immune modulation | LAM |
| WP_149291375.1 | 32.08 | VFG008136 | Immune modulation | Sulfolipid-1 biosynthesis and transport |
| WP_149291554.1, WP_149293234.1 | 30.36 - 36.36 | VFG002548 | Immune modulation | Capsule I |
| WP_149291618.1 | 31.67 | VFG001311 | Immune modulation | Capsule |
| WP_149291619.1 | 44.2 | VFG000037 | Immune modulation | LPS |
| WP_149291620.1 | 34.94 | VFG000033 | Immune modulation | LPS |
| WP_149291630.1 | 30.25 | VFG001302 | Immune modulation | Capsule |
| WP_149291632.1 | 30.24 | VFG001370 | Immune modulation | Capsule |
| WP_149291667.1 | 30 | VFG048841 | Immune modulation | Capsule |
| WP_149291728.1, WP_149292073.1 | 35.4 - 38.66 | VFG045304 | Immune modulation | LPS |
| WP_149291778.1 | 36.44 | VFG046955 | Immune modulation | LPS |
| WP_149291779.1 | 46.67 | VFG007965 | Immune modulation | PDIM |
| WP_149291856.1 | 47.3 | VFG007904 | Immune modulation | PDIM |
| WP_149291909.1 | 38.25 | VFG000964 | Immune modulation | Hyaluronic acid capsule |
| WP_149291945.1 | 39.04 | VFG047256 | Immune modulation | LPS |
| WP_149292009.1, WP_149293084.1 | 36.14 - 38.89 | VFG002190 | Immune modulation | Capsule |
| WP_149292076.1 | 35 | VFG002377 | Immune modulation | O-antigen |
| WP_149292081.1 | 33.73 | VFG000139 | Immune modulation | LPS |
| WP_149292084.1 | 36.97 | VFG001372 | Immune modulation | Capsule |
| WP_149292228.1 | 37.93 | VFG000320 | Immune modulation | LPS |
| WP_149292712.1 | 30.11 | VFG002230 | Immune modulation | LPS |
| WP_149292899.1 | 31.29 | VFG038249 | Immune modulation | PbpG |
| WP_149293225.1 | 45.85 | VFG045297 | Immune modulation | LPS |
| WP_149293296.1 | 39.36 | VFG013265 | Immune modulation | LOS |
| WP_149293417.1 | 32.77 | VFG024810 | Immune modulation | PDIM |
| WP_255583330.1 | 49.8 | VFG052100 | Immune modulation | LAM |
| WP_255584110.1 | 48.48 | VFG001303 | Immune modulation | Capsule |
| WP_255584243.1 | 37.12 | VFG002553 | Immune modulation | Capsule I |
| WP_149292879.1 | 30.07 | VFG002157 | Invasion | LpeA |
| WP_149291083.1 | 32.98 | VFG048409 | Nutritional/Metabolic factor | Ent |
| WP_149291087.1, WP_149291259.1, WP_149291332.1, WP_149291387.1, WP_149291664.1, WP_149291696.1, WP_149291749.1, WP_149292534.1, WP_149292989.1, WP_188065049.1, WP_255584114.1 | 30.51 - 39.57 | VFG001206 | Nutritional/Metabolic factor | FbpABC |
| WP_149291104.1 | 33.59 | VFG001814 | Nutritional/Metabolic factor | Nitrate reductase |
| WP_149291150.1 | 31.3 | VFG048459 | Nutritional/Metabolic factor | Ent |
| WP_149291166.1, WP_149291175.1, WP_255583540.1 | 30.8 - 37.61 | VFG048488 | Nutritional/Metabolic factor | Ent |
| WP_149291229.1 | 30.18 | VFG050016 | Nutritional/Metabolic factor | Bacillibactin |
| WP_149291342.1, WP_149293334.1 | 33.58 - 37.14 | VFG016046 | Nutritional/Metabolic factor | Pyoverdine |
| WP_149291766.1, WP_149292092.1 | 30 - 31.66 | VFG000344 | Nutritional/Metabolic factor | HitABC |
| WP_149291923.1 | 31.41 | VFG044336 | Nutritional/Metabolic factor | Sal |
| WP_149291991.1 | 32.48 | VFG001415 | Nutritional/Metabolic factor | LipF |
| WP_149292061.1 | 34.15 | VFG044083 | Nutritional/Metabolic factor | Pyoverdine |
| WP_149292397.1 | 33.82 | VFG001267 | Nutritional/Metabolic factor | Pyochelin |
| WP_149292455.1 | 33.33 | VFG000366 | Nutritional/Metabolic factor | Yersiniabactin |
| WP_149292836.1 | 38.74 | VFG015542 | Nutritional/Metabolic factor | Pyocyanin |
| WP_149293011.1 | 30.77 | VFG000365 | Nutritional/Metabolic factor | Yersiniabactin |
| WP_149293025.1 | 32.24 | VFG049994 | Nutritional/Metabolic factor | Bacillibactin |
| WP_149293150.1 | 35.45 | VFG015509 | Nutritional/Metabolic factor | Pyocyanin |
| WP_149293207.1 | 35 | VFG037344 | Nutritional/Metabolic factor | Acinetobactin |
| WP_149293228.1 | 31.92 | VFG000368 | Nutritional/Metabolic factor | Yersiniabactin |
| WP_149293258.1 | 32.75 | VFG001266 | Nutritional/Metabolic factor | Pyochelin |
| WP_149293265.1 | 40.8 | VFG047690 | Nutritional/Metabolic factor | Pyrimidine biosynthesis |
| WP_149293323.1 | 37.96 | VFG000925 | Nutritional/Metabolic factor | Enterobactin |
| WP_149293411.1 | 35.48 | VFG047564 | Nutritional/Metabolic factor | Purine biosynthesis |
| WP_149293424.1, WP_149293436.1 | 31.75 - 33.79 | VFG047582 | Nutritional/Metabolic factor | Purine biosynthesis |
| WP_188064968.1 | 34.12 | VFG010882 | Nutritional/Metabolic factor | CcmC |
| WP_255583387.1 | 31.89 | VFG000574 | Nutritional/Metabolic factor | MgtBC |
| WP_255583651.1 | 31.82 | VFG001205 | Nutritional/Metabolic factor | FbpABC |
| WP_255584199.1 | 30 | VFG047726 | Nutritional/Metabolic factor | Pyrimidine biosynthesis |
| WP_255584340.1 | 32.48 | VFG047546 | Nutritional/Metabolic factor | Biotin synthesis |
| WP_149292643.1 | 33.96 | VFG001385 | Others | MmaA4 |
| WP_149292838.1 | 34.29 | VFG001864 | Post-translational modification | Mip |
| WP_149290927.1, WP_149292338.1 | 40 - 72.95 | VFG001826 | Regulation | RelA |
| WP_149291154.1, WP_149291960.1, WP_149292868.1 | 30.09 - 69.71 | VFG001824 | Regulation | DevRS |
| WP_149291256.1 | 39.29 | VFG001414 | Regulation | WhiB3 |
| WP_149291443.1 | 61.79 | VFG001379 | Regulation | HspR |
| WP_149291959.1 | 40.38 | VFG001825 | Regulation | DevRS |
| WP_149292130.1, WP_255583475.1, WP_255583559.1 | 32.69 - 71.78 | VFG001392 | Regulation | SigE |
| WP_149292876.1 | 31.47 | VFG001406 | Regulation | IdeR |
| WP_149292950.1 | 31.49 | VFG009810 | Regulation | PhoP |
| WP_149293085.1 | 33.86 | VFG000478 | Regulation | Fur |
| WP_149293399.1, WP_149293486.1 | 35.42 - 37.8 | VFG001386 | Regulation | PhoP |
| WP_255583708.1 | 33.05 | VFG045467 | Regulation | CdpA |
| WP_149292318.1 | 42.06 | VFG037100 | Stress survival | MsrAB |
